# Dual Mechanisms of Action of a Halogenated Allyl Fatty Acid against Methicillin-Resistant *Staphylococcus aureus*

**DOI:** 10.1021/acsomega.5c07162

**Published:** 2025-11-13

**Authors:** Jazmar Villarini-Torres, Giancarlo Casillas-Vargas, Karama Shayeb, Alexis Rosado-Ortíz, Luzmarie Reyes-Vicente, Mayerli De Jesús-Vega, Derik Amely-Gavilán, Natasha Díaz-Cruz, Gil Cortés-Rodríguez, Jessica Said, Jasmin Ceja-Vega, Elizabeth Andersen, Amani Rabadi, Antonio Colom, Harry Rivera, Sunghee Lee, Nataliya Chorna, Kathleen Brundage, Néstor M. Carballeira, David J. Sanabria-Ríos

**Affiliations:** a Department of Natural Sciences, 1568 Inter American University of Puerto Rico, Metropolitan Campus, P.O. Box 191293, San Juan, Puerto Rico 00919, United States; b Medicinal Research and Applications Laboratory, 1568 Inter American University of Puerto Rico, Metropolitan Campus, P.O. Box 191293, San Juan, Puerto Rico 00919, United States; c Department of Natural Sciences and Mathematics, Inter American University of Puerto Rico, Bayamón Campus, 500 Road Dr., John Will Harris, Bayamón, PR 00957, United States; d Department of Chemistry and Biochemistry, Iona University, 715 North Avenue, New Rochelle, New York 10801, United States; e Department of Biochemistry, Medical Sciences Campus, University of Puerto Rico, P.O. Box 365067, San Juan, PR 00936, United States; f Department of Microbiology, Immunology & Cell Biology, 5631 West Virginia University P.O. Box 9177, Morgantown, West Virginia 26506, United States; g Department of Chemistry, University of Puerto Rico, Río Piedras Campus, 17 Ave Universidad STE 1701, San Juan, PR 00925, United States

## Abstract

Antimicrobial resistance in MRSA demands agents with new mechanisms. We evaluated two synthetic unsaturated fatty acids, 2-hexadecynoic acid (2-HDA) and (*Z*)-2-allyl-3-bromo-2-hexadecenoic acid (DAT-51), across clinical and reference MRSA strains. Both compounds reduced the viability in a dose-dependent manner. Fluorescence microscopy and nucleic acid leakage demonstrated rapid membrane permeabilization; flow cytometry revealed strong depolarization, and SEM showed surface lesions consistent with pore formation. DAT-51 additionally induced Lipid II accumulation and inhibited MurA, whereas lipidomics indicated the selective incorporation of 2-HDA into bacterial phospholipids. Vero-cell assays showed a low cytotoxicity at concentrations exceeding MICs. Together, the data support a dual-action paradigm: 2-HDA primarily disrupts membranes via insertion and pore formation, while DAT-51 combines moderate membrane perturbation with the inhibition of peptidoglycan biosynthesis. These findings define complementary mechanisms for uFA-based agents and identify 2-HDA and DAT-51 as promising scaffolds against drug-resistant Gram-positive pathogens.

## Graphical abstract

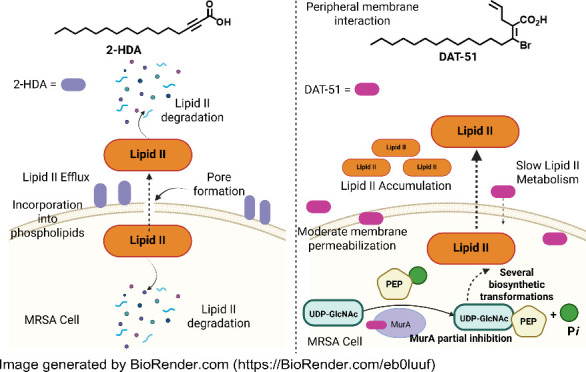

## 1 Introduction

Antimicrobial resistance (AMR) is a global public health crisis undermining decades of progress in infectious disease treatment.^1–3^ The widespread use and, in many cases, misuse of antibiotics has accelerated the emergence of multidrug-resistant organisms, creating a serious threat to global health systems. Among the most notorious antibiotic-resistant pathogens are *Staphylococcus aureus*, particularly methicillin-resistant strains (MRSA), which are associated with a broad spectrum of diseases, including skin and soft tissue infections, pneumonia, endocarditis, and sepsis.^4–6^ The Centers for Disease Control and Prevention (CDC) has classified MRSA as a serious threat, citing over 323,700 infections and more than 10,600 deaths annually in the United States.^7^ This challenge is exacerbated by the declining number of new antibiotics in the development pipeline, especially those targeting resistant Gram-positive bacteria.^8,9^

In response to this critical need, there is growing interest in exploring nontraditional antibacterial agents with novel mechanisms of action. Fatty acids (FA), mainly unsaturated fatty acids (uFA), have emerged as promising candidates due to their broad-spectrum antimicrobial activity and broad biological functions.^10–14^ These amphipathic molecules are naturally found in mammalian skin, mucosal barriers, and plant defense systems, where they contribute to innate immunity by inhibiting the growth of pathogenic microbes.^15–17^ Several studies have demonstrated that uFA exhibit bactericidal properties, often attributed to their ability to disrupt bacterial membranes or interfere with essential physiological processes.^18–22^ However, most existing literature focuses on naturally occurring uFA, which is often limited in terms of availability, metabolic stability, or chemical versatility.

Synthetic uFA, by contrast, offers several advantages as antibacterial agents. Their chemical structures can be precisely tailored to enhance potency, selectivity, and metabolic stability.^23,24^ Moreover, synthetic routes to obtain these compounds are relatively straightforward, making them attractive candidates for structure–activity relationship (SAR) studies and mechanistic investigations.^25,26^ In recent years, synthetic uFA containing double or triple bonds, halogenated substituents, or branching patterns have demonstrated potent activity against Gram-positive and Gram-negative bacteria, including multidrug-resistant strains.^27–35^ These observations have stimulated interest in understanding how specific structural features of uFA contribute to their antibacterial effects.

Despite promising *in vitro* findings, the underlying mechanisms by which synthetic uFA exert its bactericidal activity remain poorly defined. It is generally accepted that membrane perturbation plays a central role in the antibacterial action of these compounds.^22,36,37^ However, it is not yet clear whether this effect results solely from nonspecific membrane destabilization or whether it involves more specific interactions with key components of the bacterial envelope, such as peptidoglycan (PG) biosynthetic enzymes or intermediates such as Lipid II. Some uFAs have been proposed to mimic the activity of cationic antimicrobial peptides by inserting into the bacterial membrane and forming pores.^14,22^ In contrast, others may interfere with the assembly or function of the cell wall.^37,38^

Additional questions remain regarding incorporating exogenous uFA into bacterial membranes and how such incorporation might alter the membrane composition, biophysical properties, or susceptibility to stress. Moreover, certain uFAs inhibit the expression of bacterial virulence factors,^39–41^ reduce plasmid conjugation,^42–45^ or modulate the redox balance within the cell,^46–48^ suggesting that multiple overlapping mechanisms may be at play. These complexities underscore the need for systematic studies that elucidate the interactions between synthetic uFA and bacterial targets at both the molecular and cellular levels.

Considering these knowledge gaps, this study focuses on evaluating the mechanistic basis of the antibacterial activity of two synthetic uFA: 2-hexadecynoic acid (2-HDA, Figure 1), an acetylenic fatty acid containing a triple bond at C-2, and (*Z*)-2-allyl-3-bromo-2-hexadecenoic acid (DAT-51, Figure 1), a halogenated fatty acid with a double bond at C-2 and a bromine atom at C-3.

**Figure 1:**
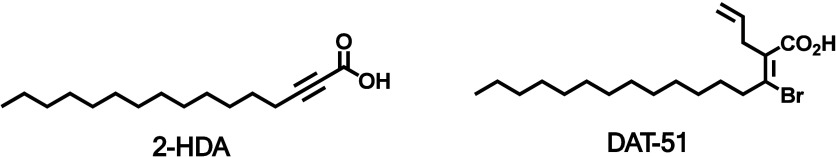
Chemical structures of the triple-bonded 2-HDA and the double bonded DAT-51. The synthesis and chemical characterization of both synthetic uFA was reported elsewhere.^27,32,33^

Both compounds were previously reported to display potent activity against MRSA, including ciprofloxacin-resistant strains, and exhibit low toxicity to mammalian cells.^27,32,33^ Given their promising antibacterial profiles, 2-HDA and DAT-51 are ideal candidates for mechanistic investigations to uncover how synthetic uFA decreases bacterial viability. Specifically, this research seeks to determine whether these compounds exert their activity through membrane disruption, inhibition of peptidoglycan biosynthesis, or a combination of both.

By clarifying these mechanisms, this study aims to provide a scientific foundation for the rational design of the next generation of uFA with enhanced antibacterial properties. Furthermore, advancing our understanding of how synthetic fatty acids interact with resistant bacterial targets may contribute to developing innovative therapeutic strategies to combat AMR. The findings reported herein are particularly relevant given the urgent need for novel, effective, and accessible antibacterial agents to overcome resistance in MRSA and other clinically significant pathogens.

## 2 Materials and Methods

### 2.1 Synthesis of 2-HDA and DAT-51

The synthesis of 2-HDA and DAT-51 was performed following previously reported procedures with minor modifications.^27,32,33^ As mentioned above, the procedures described a straightforward multistep organic synthesis that included carboxylation reactions and palladium-catalyzed allyl halide addition steps. As described in the original procedures, the purity and structural identities of the final products were confirmed by NMR, FT-IR, and GC-MS.

### 2.2 Microorganisms and Eukaryotic Cells

For microbiological assays, the following bacterial strains were used as model organisms: *Staphylococcus aureus* methicillin-resistant strain XIII (MRSA XIII), a clinical isolate obtained from a hospital-associated infection and confirmed to be resistant to both β-lactam antibiotics and ciprofloxacin, and MRSA ATCC 43300. MRSA ATCC 43300 was obtained from the American Type Culture Collection (ATCC, Manassas, VA, USA), while MRSA XIII was maintained in our laboratory collection. Vero cells derived from the kidney epithelial tissue of the African green monkey (*Chlorocebus sabaeus*) were employed as the eukaryotic model for cell culture assays. These cells were also acquired from ATCC (CCL-81).

### 2.3 Bacterial Quantification by CFU Counting

Single MRSA XIII colonies were individually cultured in Tryptic Soy Broth (TSB) and incubated at 37 °C with constant agitation (180 rpm) for 18–22 h. Overnight cultures were diluted 1:200 in fresh sterile TSB and further incubated under identical conditions for 3 h until reaching an OD_600_ of approximately 1.0. Bacterial suspensions were then mixed in defined volumetric ratios (4:1, 3:2, 2:3, 1:4, and 1:9), as described by Campbell et al.^49^ and subjected to serial 10-fold dilutions ranging from 10^–2^ to 10^–6^ using standard aseptic techniques. Aliquots (100 μL) from dilutions 10^–4^ to 10^–6^ were plated onto tryptic soy agar (TSA) and incubated for 18–20 h at 37 °C. Colonies were counted, and plates yielding 30–300 CFUs were considered suitable for quantification. Bacterial density (CFU/mL) was calculated accordingly.^49^ A calibration curve was generated by plotting CFU/mL versus OD_600_. Linear regression analysis was applied to derive the best-fit equation and coefficient of determination (*R* ^2^), establishing the correlation between optical density and viable bacterial count (see Supporting Information section, Figure S1).

### 2.4 Determination of MRSA Viability under uFA Treatment

Antibacterial susceptibility assays were performed using protocols routinely employed in our laboratory and previously reported in the literature.^27,29,31–33^ Briefly, single colonies of MRSA ATCC 11493 and ATCC 43300 were cultured in 10 mL of tryptic soy broth (TSB) at 37 °C with agitation (180 rpm) for 18–22 h. Stock solutions of either 2-HDA or DAT-51 were prepared in 100% DMSO and serially diluted in sterile TSB. Each dilution (100 μL) was dispensed into flat-bottom 96-well microplates preinoculated with 10 μL of TSB containing 4–5 × 10^5^ CFU. Plates were incubated at 37 °C for 18–20 h. Bacterial viability was further assessed via the MTT assay as described by Sanabria-Ríos et al.^34^ Reduction of MTT to formazan by metabolically active cells was quantified at 570 nm by using a Varioskan LUX multimode microplate reader (Thermo Scientific). Control samples without treatment and containing sterile TSB were included for comparison, and the minimum inhibitory concentration (MIC) was defined as the lowest concentration that inhibited visible growth.

### 2.5 Assessment of Bacterial Membrane Integrity Using Fluorescein Staining

MRSA XIII and ATCC 43300 were cultured in TSB at 37 °C with shaking to mid log following an overnight preculture as described by Sanabria-Ríos et al.^27,32^ Cell suspensions were treated for 4 h with 2-HDA or DAT-51 at 4× MIC (final 1% DMSO vehicle; nisin as positive control). After treatment, cells were pelleted (5 min, 4 °C), washed 3 times with ice-cold 1× PBS, and costained with DAPI (1 μg/mL) and propidium iodide (PI, 1 μg/mL) for 20 min on ice in the dark as described in the literature.^50^ Stained suspensions (1.8–10 μL) were mounted under a coverslip and imaged on a MOTIC AE31E fluorescence microscope (Motic, Germany) using the DAPI filter set (Ex 350–405 nm/Em 450–480 nm (blue)) and the PI/TRITC filter set (Ex 535–561 nm/Em 600–650 nm). Exposure and gain were identical across conditions, and only the DAPI and PI channels were acquired. Imaging was performed qualitatively; representative fields for each condition are shown. Graphs showing MIC values for nisin in MRSA XIII and MRSA ATCC 43300 are shown in the Supporting Information (Figures S2 and S3, respectively).

### 2.6 Detection of Membrane Disruption via Nucleic Acid Leakage

Membrane disruption and nucleic acid leakage were evaluated by measuring the absorbance at 260 nm (A_260_) in MRSA XIII following treatment with either 2-HDA or DAT-51. MRSA XIII suspension (10^7^ CFU/mL, 10 mL) was treated with synthetic uFA at its MIC value corresponding to each test compound. In this experimental design, 1% DMSO, nisin, and palmitoleic acid were controls. At defined time points (0, 20, 40, 60, and 80 min), 1 mL aliquots were collected and centrifuged (2,000 × *g*, 10 min). The absorbance of the supernatant was recorded at 260 nm using a Genesys 10S UV–vis spectrophotometer (Thermo Fisher Scientific, Cambridge, UK) to quantify extracellular DNA. Graph bars showing the MIC for both nisin and palmitoleic acid in MRSA XIII are shown in Figures S2 and S4, respectively.

### 2.7 Assessment of Membrane Potential by Flow Cytometry

Membrane potential was assessed using the BacLight Bacterial Membrane Potential Kit (Thermo Fisher Scientific) according to the manufacturer’s protocol. MRSA XIII cells (2 × 10^6^ CFU/mL) were incubated with either 1% DMSO (vehicle control) or uFA treatment (4× MIC). Samples were stained with DiOC_2_(3) (3 mM) and TO-PRO-3 iodide (100 μM) in filtered PBS. Depolarized controls were prepared using carbonyl cyanide *m*-chlorophenylhydrazone (CCCP, 500 μM). After 30 min of incubation in the dark at room temperature, cells were centrifuged (13,300 rpm, 30 s), washed twice with PBS, and fixed with 4% paraformaldehyde for 15 min at 4 °C. Fixed samples were washed, stored at 4 °C, and analyzed using a 4 laser LSR Fortessa flow cytometer (BD Biosciences) equipped with FSC PMT and with FACSDiva software version 8.0. A minimum of 10,000 bacteria were analyzed per sample. Flow cytometry data analysis was done using FCS Express version 6 or 7 (De Novo Software). Standard curve correlating OD_600_ and viable cell count can be accessed in the Supporting Information section (Figure S1).

### 2.8 Scanning Electron Microscopy (SEM) Analysis of uFA-Treated MRSA

Morphological changes in MRSA cells treated with uFA (4× MIC) and untreated controls were analyzed by scanning electron microscopy (SEM), following a modified protocol based on Lv et al.^51^ After incubation at 37 °C with shaking (180 rpm) for 18–20 h, samples were centrifuged (2,500 × *g*, 10 min), washed with 0.22 μm-filtered PBS, and fixed in 2.5% glutaraldehyde at room temperature (30 min, dark) followed by overnight fixation at 4 °C. Fixed cells were washed with 0.2 M sodium phosphate buffer (pH 7.2), dehydrated through graded ethanol (30% to 100%), and centrifuged at each step. Samples were lyophilized overnight (Millrock Benchtop Freeze-Dryer 210) and stored at 4 °C. SEM characterization was performed by using a Phenom XL desktop instrument operated at an accelerating voltage of 10 kV. Both secondary electron (SE) and backscattered electron (BSE) detectors were employed to obtain surface morphology and contrast, respectively. To minimize charging and improve image quality, samples were gold-coated by using a Denton Desk V sputter coater, resulting in an estimated coating thickness of approximately 20–40 nm. A working distance of approximately 6.9 mm was maintained, and images were acquired at magnifications ranging from 20,000× to 50,000×. Representative micrographs are provided in the Supporting Information (Figures S5–S14).

### 2.9 Detection of Lipid II Accumulation Induced by Synthetic Fatty Acids

Lipid II was isolated and biotinylated using the protocol described by Qiao et al.^52^ Briefly, membrane-associated peptidoglycan precursors were obtained from MRSA XIII cultures treated with 2-HDA, DAT-51 (each at 4× MIC), or 1% DMSO as a vehicle control. Biotinylation of Lipid II was carried out using purified *S. aureus* PBP4 (GenScript, Piscataway, NJ, USA) in the presence of biotinylated d-lysine (BDL), allowing site-specific labeling of Lipid II via transpeptidase-mediated D-amino acid exchange. Biotinylated samples were then subjected to thin-layer chromatography (TLC) using the method described by Pazos et al.,^53^ with modifications. Biotinylated samples were directly spotted onto silica gel TLC plates and developed using a pre-equilibrated chamber containing a solvent system composed of acetonitrile, ethyl acetate, isopropanol, and water in a 4.25:1:2.5:3 ratio (v/v/v/v). Streptavidin-HRP (1:1,000 v/v, Sigma-Aldrich, St. Louis, MO, USA) was used to detect biotin-labeled Lipid II. After development, TLC plates were dried and placed in a sealed iodine vapor chamber for visualization. Band intensities were quantified via densitometric analysis using ImageJ (NIH, Bethesda, MD, USA) to assess Lipid II accumulation.

### 2.10 Assessment of MurA Inhibition by Synthetic Fatty Acids

Enzymatic assays targeting *S. aureus* MurA were performed following established protocols described in the literature, with minor modifications.^54–56^ MurA catalyzes the transfer of enolpyruvate from phosphoenolpyruvate (PEP) to UDP-*N*-acetylglucosamine (UDP-GlcNAc), generating inorganic phosphate as a byproduct. Inorganic phosphate release was quantified using a malachite green-based assay (Sigma-Aldrich, St. Louis, MO, USA). Recombinant MurA was obtained from GenScript (Piscataway, NJ, USA).

Reactions were conducted in a HEPES buffer at pH 7.8, which was supplemented with KCl and MgCl_2_. Each reaction included 1.0 μM MurA, 200 μM UDP-GlcNAc, 100 μM PEP, and either 2-HDA, DAT-51, linoleic acid, palmitoleic acid, or phosphomycin at a concentration of four times MIC (4× MIC) (MIC values from MRSA XIII of nisin, palmitoleic acid, linoleic acid, and phosphomycin are available in the Supporting Information section, see Figures S2, S4, S15, and S16, respectively). A vehicle control consisting of 1% DMSO was also included. Reaction mixtures were transferred to a 96-well plate and incubated for 30 min at room temperature to allow the reaction to proceed. After that, the malachite green working reagent was added to each well. The mixture was then incubated for an additional 30 min at room temperature. Absorbance was measured at a wavelength of 620 nm using an accuSkan FC microplate reader (Fisher Scientific). All reactions were performed in six biological replicates to ensure reproducibility.

### 2.11 Molecular Docking Analysis

Molecular docking was performed using the SwissDock web server (http://old.swissdock.ch), and docking calculations were performed using the AutoDock Vina 1.2.5 algorithm.^57^ Ligands tested included 2-HDA, DAT-51, linoleic acid, palmitic acid, and fosfomycin (positive control). Ligand structures were submitted in SMILES notation. The target protein structure of *Escherichia coli* MurA was retrieved from the Protein Data Bank (PDB ID: 3KR6). Before docking, the suitability of this structure as a model for *S. aureus* MurA was assessed by performing a pairwise sequence alignment using the BLASTp tool against the *S. aureus* MurA sequence (UniProt ID: Q2FWD4).^58,59^ Blind docking was conducted using SwissDock’s default parameters. Binding modes were ranked by SwissDock scores,^60,61^ and the top-ranked poses were analyzed using the Maestro visualization platform (Schrödinger, LLC) to examine the 3D structure of the ligand-MurA complexes. This workflow enabled efficient prediction of potential ligand-MurA interaction sites.

### 2.12 Determination of Fatty Acid Composition by GC-MS

To investigate whether the antibacterial effect of synthetic uFA involves their incorporation into the *S. aureus* membrane, we analyzed the FA composition using column chromatography followed by gas chromatography–mass spectrometry (GC-MS), based on the method described in the literature.^32,62^ MRSA XIII was cultured in tryptic soy broth (TSB) at 37 °C for 18–20 h in the presence or absence of either 2-HDA or DAT-51 at their respective MIC values. After incubation, bacterial cells were harvested by centrifugation at 5,000 rpm for 5 min at 4 °C (Sorvall ST 16R, Thermo Scientific) and washed three times with cold 1× PBS to eliminate medium residues.

Cell disruption was performed using mechanical homogenization with a Bead Ruptor Elite (OMNI International) at 4.00 m/s for two cycles of 10 min each followed by 20 min of sonication in a Fisher Brand ultrasonic cleaner (FB11201) operating at 37 kHz and 100% power (390 W) at room temperature. The lysed samples were centrifuged at 17,000 × *g* for 20 min (Sorvall Legend Micro 17, Thermo Scientific) to separate the soluble lipid fraction. Lipid components from the supernatant were extracted using silica gel column chromatography (silica gel 60, 70–230 mesh). Diethyl ether was used as the mobile phase to selectively elute free fatty acids, including any unbound or nonincorporated synthetic uFA. At the same time, phospholipids remained bound or were collected separately using methanol elution if required. Organic fractions were evaporated to dryness under reduced pressure by using a Büchi Rotavapor R-114. Dried lipid fractions were refluxed in 3 mL of methanol containing 150 μL of 12 M hydrochloric acid at 85 °C to derivatize FA to its FA methyl esters (FAMEs) for 3 h. After cooling, the methanol was evaporated, and the samples were further dried by lyophilization at −80 °C (Millrock Technology freeze-dryer). FAMEs were subjected to GC-MS analysis using a Shimadzu GC/MS-QP2010 equipped with a FAMEWAX capillary column (30 m × 0.32 mm i.d., 0.25 μm film thickness, Restek) and an autoinjector (AOC-20i).

The chromatographic method involved an initial oven temperature of 130 °C, ramped at 4 °C/min to a final temperature of 250 °C. Helium served as the carrier gas with a 15:1 split ratio. Mass spectra were acquired in full scan mode from 50 to 600 amu under electron impact ionization (70 eV) with an ion source temperature of 200 °C. Both injector and detector temperatures were maintained at 250 °C. Identification of 2-HDA or DAT-51 was based on comparison of their molecular ion, base peak, fragmentation pattern, retention time, and equivalent chain length (ECL). Data were expressed as the percentage of relative abundance of total FA, and all experiments were performed in biological triplicate.

### 2.13 MTT-Based Cytotoxicity Evaluation of 2-HDA

African green monkey kidney epithelial cells (Vero, ATCC CCL-81) were seeded at a density of 1 × 10^4^ cells/well in 96-well plates and cultured in Dulbecco’s modified Eagle medium (DMEM; Gibco, Thermo Fisher Scientific, Waltham, MA, USA) supplemented with 10% fetal bovine serum (FBS; HyClone, Cytiva, Marlborough, MA, USA). After 24 h of incubation at 37 °C in a humidified atmosphere with 5% CO_2_ to allow cell adhesion, cells were treated with serial dilutions of 2-HDA (prepared in complete DMEM containing 1% DMSO) and incubated for 24 h. Cytotoxicity was assessed using the MTT assay. A 0.5 mg/mL MTT solution was added to each well and incubated for 2 h at 37 °C. Formazan crystals were solubilized in 100 μL of 100% DMSO, and absorbance was measured at 570 nm using a Varioskan LUX multimode microplate reader (Thermo Scientific). Cell viability was expressed as a percentage relative to DMSO-treated controls. Experiments were performed in six independent biological replicates. The cytotoxicity of 2-HDA was assessed by comparing bar graphs of cell viability against those of the untreated control (1% DMSO). Graphs were generated using GraphPad Prism based on normalized absorbance data obtained from the MTT assay.

### 2.14 Water Permeability Measurements

1,2-Dioleoyl-*sn*-glycero-3-phosphocholine (18:1 (Δ^9^ *cis*) PC, DOPC) (Avanti Polar Lipids, Inc.) was used as supplied in chloroform and stored at −20 °C. Squalene (SqE) (Sigma-Aldrich) was stored at 2–8 °C. Dried lipid films were prepared by evaporating chloroform under inert gas followed by overnight vacuum drying, and then dissolved in SqE at 5 mg/mL. 2-HDA was codissolved with DOPC in chloroform before solvent removal. As previously described, water permeability was measured using a droplet interface bilayer (DIB) protocol.^63,64^ A DIB forms when two lipid-coated aqueous microdroplets contact, creating a bilayer that closely mimics the structure of cell membranes.^65^ In this method, two lipid-coated aqueous microdroplets (∼100 μm diameter), one containing pure water and the other 0.1 M NaCl, are brought into contact in SqE containing DOPC or DOPC with 2-HDA, forming a DIB. Osmolarity was verified before use. Water transport across the DIB, driven by the osmotic gradient, is quantified by monitoring changes in droplet diameter in real time using a micropipet manipulation station (Narishige) integrated with an inverted microscope (Nikon Eclipse Ti–S, halogen lamp) and a camera (Andor Zyla sCMOS). Experiments were performed at 30 °C in a temperature-controlled microchamber, and changes in droplet size were analyzed using custom image analysis software. Each data point represents the mean of at least 10 independent measurements, with standard deviations shown as error bars.

### 2.15 Differential Scanning Calorimetry (DSC)

Multilamellar vesicles (MLVs) were prepared by hydrating dried lipid films with pure water to a final concentration of 16 mg/mL followed by vortexing and 30 min bath sonication. DSC measurements were performed on a TA Q2000 using aqueous MLV dispersions of DOPC or DOPC with 2-HDA. Approximately 15 μL of each sample was hermetically sealed and cycled between −40 and 0 °C at 5 °C/min under a 50 mL/min nitrogen purge. Each sample underwent three heating and cooling cycles to assess hysteresis with reproducible results. Reported values are averages from two independent samples. Main phase transition temperature (*T*_m_) and enthalpy (Δ*H*) were determined by using TA Universal Analysis software.

### 2.16 Confocal Raman Microspectroscopy

Hydrated lipid suspensions underwent seven freeze–thaw cycles. All aqueous solutions were prepared using deionized water (18.2 MΩ·cm). Raman microspectroscopy was performed by using an XploRA INV (Horiba) inverted confocal Raman microscope equipped with a 532 nm laser and a cooled CCD detector. Lipid suspensions (10–20 μL), prepared by freeze–thaw cycles, were deposited on cleaned glass coverslips and dried at ∼30 °C to form supported bilayers. Spectra were acquired at room temperature using a 10× objective and an 1800 lines/mm grating. Two independent samples were analyzed across multiple regions, and the results were averaged. Data were acquired and processed with LabSpec 6 software.

### 2.17 Biostatistical Analysis

All biological assays were performed with at least six independent biological replicates to ensure statistical robustness. Data was analyzed using one-way ANOVA and Dunnett’s test to compare each treatment to the control group, with no treatment containing only 1% DMSO vehicle. Results are presented as the mean ± standard error of the mean (SEM). Statistical analyses and graphical representations were conducted using GraphPad Prism version 8.3 (GraphPad Software, Bishops Stortford, UK). A *p*-value <0.05 was considered statistically significant.

## 3 Results

### 3.1 Synthetic uFA 2-HDA and DAT-51 Inhibit MRSA Proliferation in a Concentration-Dependent Manner

To evaluate the antibacterial effects of synthetic unsaturated fatty acids (uFA), we treated both MRSA XIII and MRSA ATCC 43300 with increasing concentrations of 2-HDA (0.06–31.3 μg/mL) and DAT-51 (1.95–1000 μg/mL) for 18–20 h at 37 °C. Bacterial viability was assessed using the MTT assay, which quantifies metabolic activity by measuring the reduction of MTT to formazan at 570 nm.

As shown in Figure 2A, exposure of MRSA XIII to 2-HDA resulted in a sharp decline in viability, starting at 1.0 μg/mL, with the proliferation of viable cells dropping below 40% at 2.0 μg/mL and continuing to decrease at higher concentrations. For DAT-51 (Figure 2B), the reduction in proliferation was more gradual, with the viability remaining relatively steady from 0 to 31.3 μg/mL and then declining significantly at concentrations above 62.5 μg/mL.

**Figure 2:**
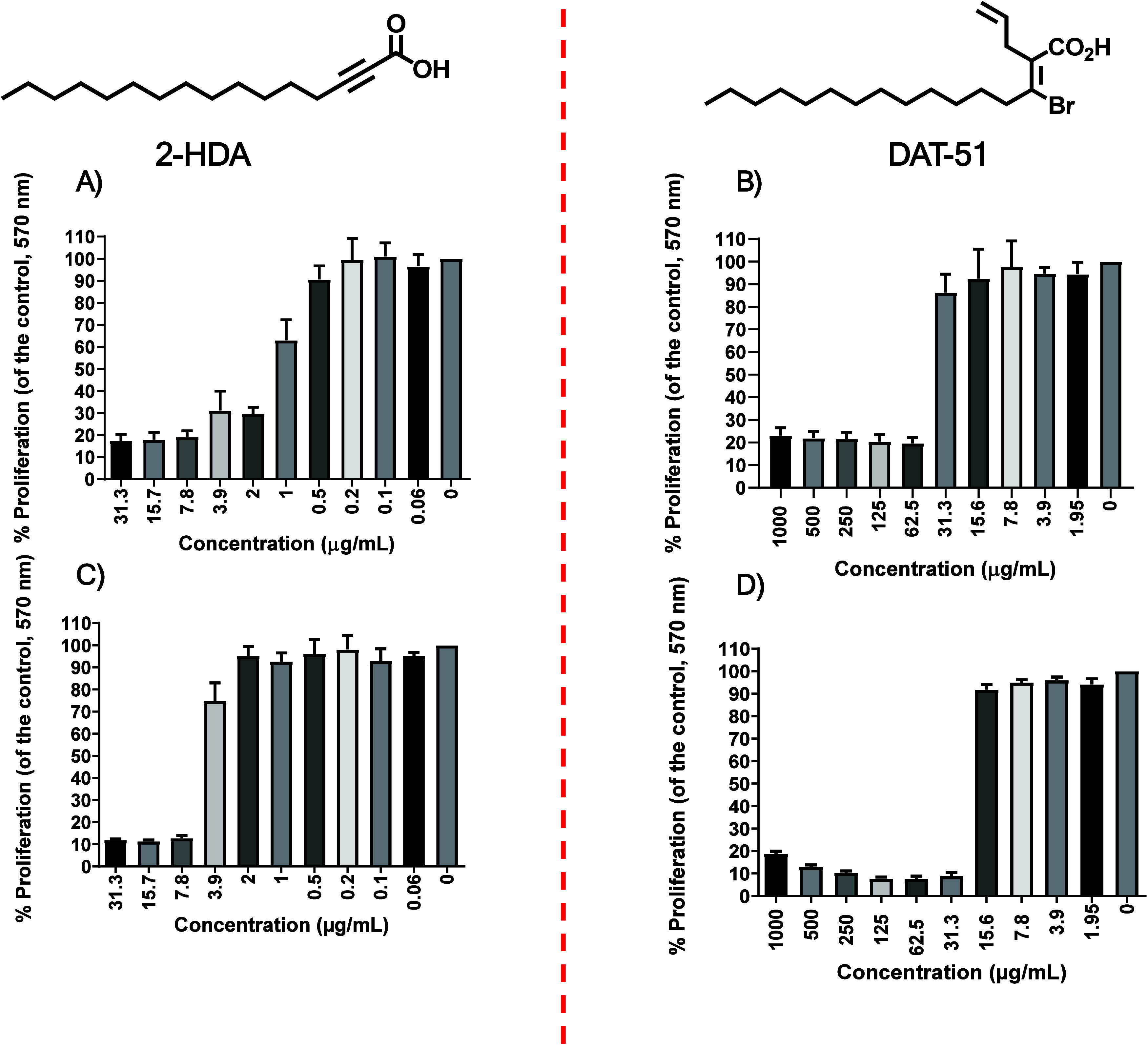
Antibacterial activity of 2-HDA and DAT-51 against MRSA assessed by the MTT assay. Cell viability was evaluated in *S. aureus* strains MRSA XIII (A, B) and MRSA ATCC 43300 (C, D) following exposure to increasing concentrations of synthetic uFA 2-HDA (A, C) and DAT-51 (B, D). Viability was measured using the MTT assay, which quantifies the reduction of MTT to formazan at 570 nm as an indicator of metabolic activity. Bacterial cultures were incubated for 18–20 h at 37 °C. Bars represent the mean ± SEM from six independent biological replicates.

A similar trend was observed in the MRSA ATCC 43300 strain. As shown in Figure 2C, treatment with 2-HDA caused a reduction in metabolic activity beginning at 3.9 μg/mL, with viability falling below 10% at 7.8 μg/mL. In contrast, treatment with DAT-51 (Figure 2D) led to a significant decrease in cell viability starting at 31.3 μg/mL, reaching ≤ 20% at the highest tested concentration (1,000 μg/mL). These findings confirm that both 2-HDA and DAT-51 inhibit MRSA proliferation in a concentration-dependent manner. However, 2-HDA consistently exhibited greater potency than DAT-51 in both clinical and reference strains.

### 3.2 Synthetic Fatty Acids Compromise MRSA Membrane Integrity

#### 3.2.1 Fluorescence Microscopy (DAPI/PI)

Representative DAPI/PI images show treatment-dependent membrane effects in MRSA XIII and MRSA ATCC 43300 (Figures 3 and 4). All panels were acquired with identical exposure/gain using only DAPI and PI. In the 1% DMSO panels, only a small subset of cells shows detectable DAPI labeling. At the same time, PI is absent, a pattern expected for untreated Gram-positive cells under our low-stringency staining (1 μg/mL, 20 min on ice) and fixed acquisition settings; the absence of PI indicates intact membranes. By contrast, nisin yields widespread PI uptake with magenta merges. 2-HDA increases the fraction of PI-positive cells in both strains with a more substantial effect in MRSA XIII and a moderate effect in MRSA 43300. DAT-51 produces PI staining broader than that of 2-HDA, particularly in MRSA XIII, with a minor but apparent increase in MRSA ATCC 43300.

**Figure 3:**
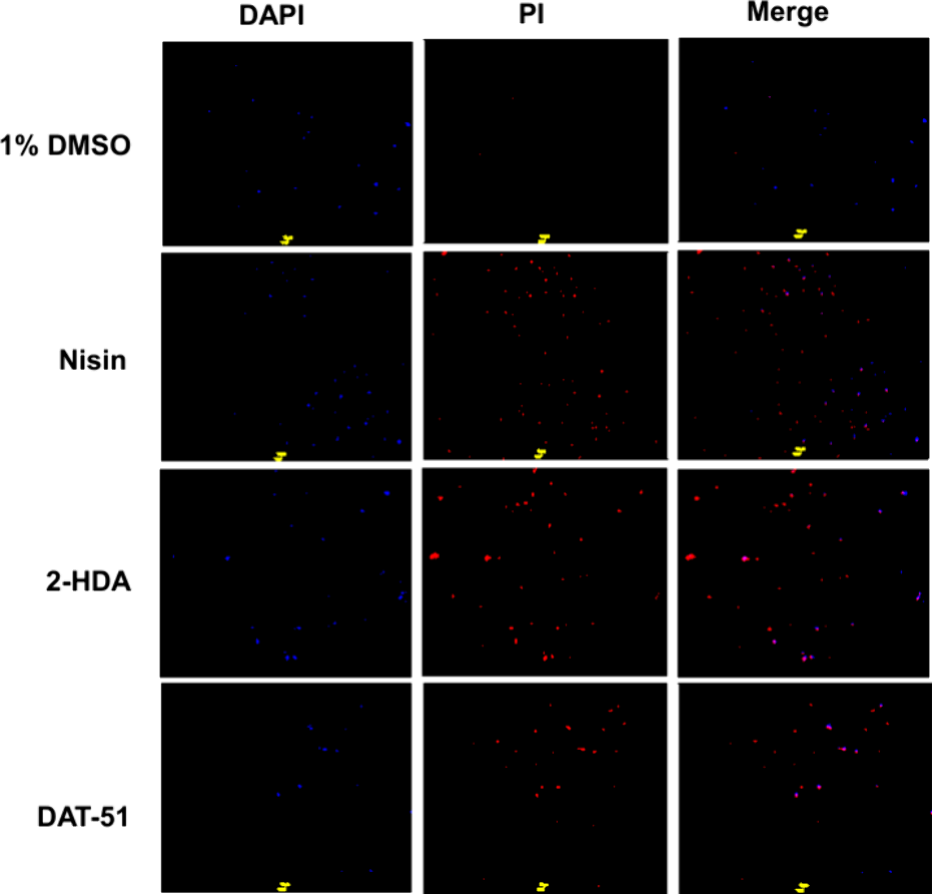
DAPI/PI live–dead microscopy of MRSA ATCC 43300 after exposure to 2-HDA and DAT-51. Representative fields acquired with identical exposure using only DAPI (blue) and PI (red) channels. Conditions: 1% DMSO, nisin (positive control), 2-HDA, and DAT-51 (each 4× MIC, 4h). Cells were stained simultaneously with DAPI and PI (1 μg/mL each, 20 min on ice, in the dark) and imaged on a MOTIC AE31E microscope by using DAPI and PI/TRITC filter sets. Scale bar: 10 μm.

**Figure 4:**
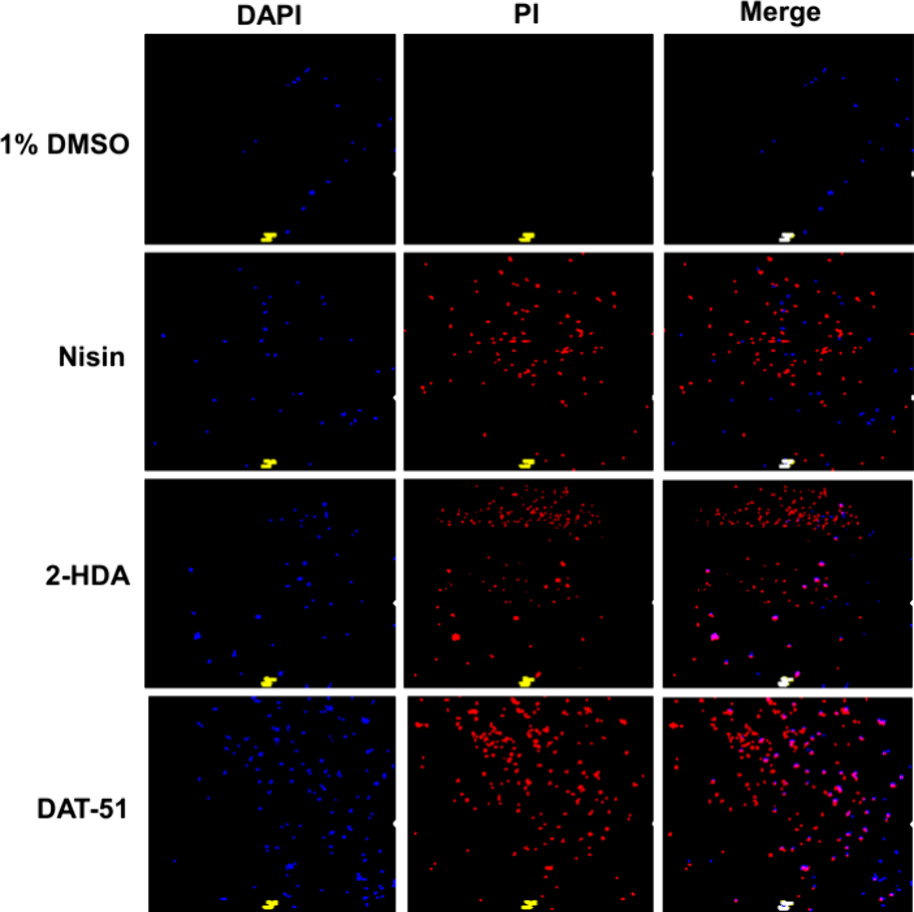
DAPI/PI live–dead microscopy of MRSA XVIII after 2-HDA and DAT-51 exposure. Representative fields were acquired with identical exposure/gain using only DAPI (blue) and PI (red) channels. Conditions: 1% DMSO, nisin (positive control), 2-HDA, and DAT-51 (each 4× MIC, 4 h). Cells were stained simultaneously with DAPI and PI (1 μg/mL each, 20 min on ice, in the dark) and imaged on a MOTIC AE31E microscope using DAPI and PI/TRITC filter sets. Scale bar = 10 μm.

#### 3.2.2 Kinetic Membrane-Permeability Assay

In a separate set of experiments (*n* = 4), following the kinetic protocol of Xu et al.,^50^ we monitored the time-resolved uptake of an impermeant DNA dye using TO-PRO-3 iodine (far-red) in place of SYTOX Green. This choice minimized Gram-positive autofluorescence on our plate reader, yielding a stable, linear signal with our optics/filters. Traces were normalized to 0% = 1% DMSO and 100% = 70% ethanol. All conditions (1–4× MIC of either 2-HDA or DAT-51) exhibited a rapid rise at ∼10–15 min followed by stable plateaus up to 60 min (Figure 5). For 2-HDA, MRSA XIII increased with dose up to ∼2–3× MIC (Figure 5A, 1× ≈ 30–45%, 2× ≈ 70–80% [max], 3× ≈ 60–70%), while 4× remained below 3× (30–40%). In MRSA 43300 (Figure 5B,D), all doses plateaued at ∼50–70% ethanol, with a 1× MIC comparable to or slightly above 2–4× MIC. For DAT-51, MRSA XIII (Figure 5C) exhibited a clear dose–response relationship from 1× to 3× MIC, with modest attenuation at 4× MIC; MRSA ATCC 43300 increased with the dose and reached plateaus similar to those of MRSA XIII.

**Figure 5:**
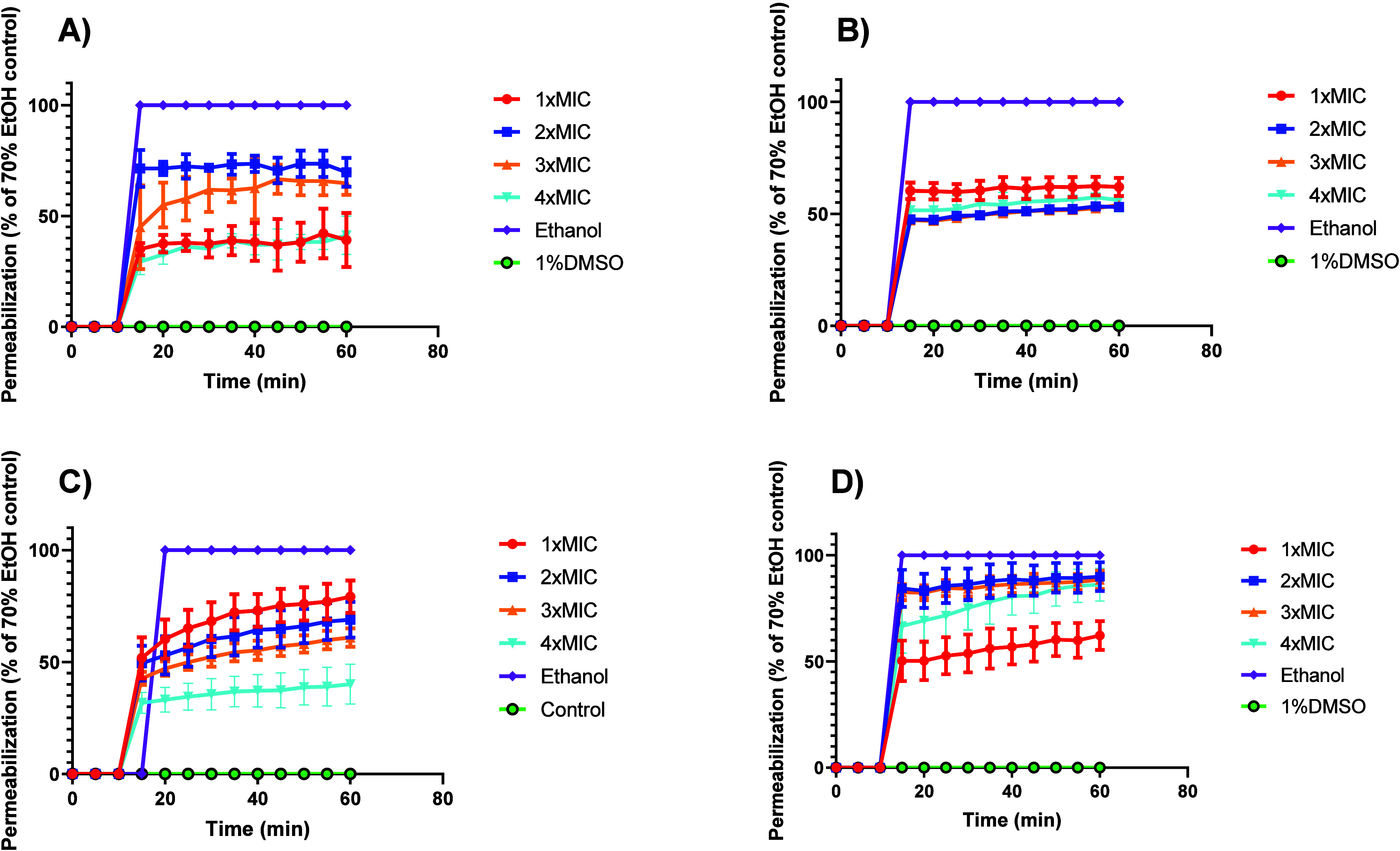
2-HDA and DAT-51 trigger rapid, dose- and strain-dependent membrane permeabilization in MRSA. MRSA cultures were treated with each compound at 1×, 2×, 3×, or 4× MIC. Time-resolved permeability was monitored with TO-PRO-3 iodine and normalized to 0% = 1% DMSO and 100% = 70% ethanol. All traces show a rapid rise (∼10–15 min) followed by stable plateaus to 60 min. (A) 2-HDA, MRSA XIII: maximal plateaus at 2–3× MIC; 4× MIC falls below 3× MIC. (B) 2-HDA, MRSA ATCC 43300: similar plateaus (∼50–70% EtOH) across doses with 1x MIC ≥ 2–4× MIC. (C) DAT-51, MRSA XIII: strongest response at 1× MIC with stepwise decreases at higher doses. (D) DAT-51, MRSA ATCC 43300: permeabilization increases with dose, approaching ∼ 60–70% of the ethanol control at 3–4× MIC. Data are mean ± SEM, *n* = 4 independent measurements per condition.

### 3.3 Synthetic uFAs Induce Time-Dependent DNA/RNA Leakage in MRSA XIII

The extent of intracellular nucleic acid release in MRSA XIII was evaluated over time following treatment with synthetic (2-HDA, DAT-51) and the naturally occurring (palmitoleic, palmitic) FA, as well as nisin. As shown in Figure 6, exposure to 2-HDA resulted in the highest DNA/RNA leakage across all time points, with normalized absorbance values significantly elevated as early as time 0. DAT-51 and nisin also caused increased leakage relative to the DMSO control, particularly at 20–60 min. Notably, palmitoleic acid showed statistically significant increases at 60 and 80 min, whereas palmitic acid did not elicit significant effects throughout the assay period.

**Figure 6:**
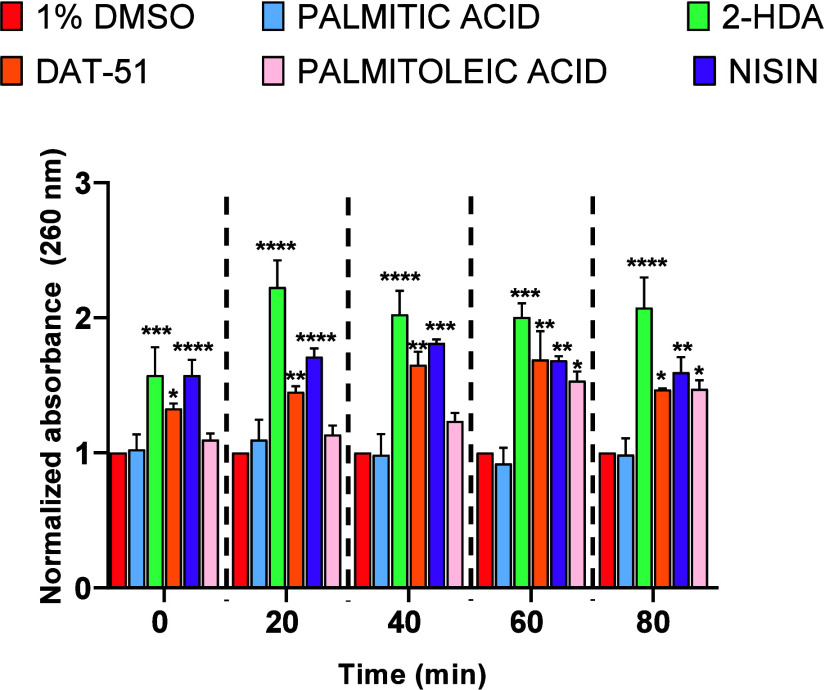
Synthetic unsaturated FA induce DNA/RNA leakage in MRSA XIII over time. Leakage of intracellular nucleic acids was evaluated in MRSA XIII cultures treated with synthetic uFA (2-HDA, DAT-51), natural-occurring FA (palmitoleic or palmitic acids), or nisin, each at their respective MICs, for up to 80 min. Absorbance at 260 nm was measured to quantify extracellular DNA/RNA release and normalized to the untreated control (1% DMSO). Data represents SEM from six independent biological replicates. One-way ANOVA was performed at each time point to assess statistical significance (*p* < 0.05) versus the DMSO control. The MIC values for nisin and palmitoleic acid in MRSA XIII are presented, respectively, in the bar graphs in the Supporting Information section (Figures S2 and S4).

### 3.4 Synthetic Fatty Acids Induce Membrane Depolarization in MRSA XIII

To assess whether the antibacterial activity of 2-HDA and DAT-51 involves membrane depolarization, MRSA XIII cells were treated with each compound at 4× MIC and analyzed using the membrane-potential-sensitive dye DiOC_2_(3). CCCP (500 μM), a known protonophore, served as the positive control for depolarization, while 1% DMSO was used as the vehicle control. Following staining, samples were analyzed by flow cytometry to quantify the ratio of red to green fluorescence (PE-Texas Red/FITC), which correlates with membrane potential.

As shown in Figure 7, CCCP (Figure 7B) treatment drastically reduced the population with a high membrane potential (6.05%), confirming depolarization.

**Figure 7:**
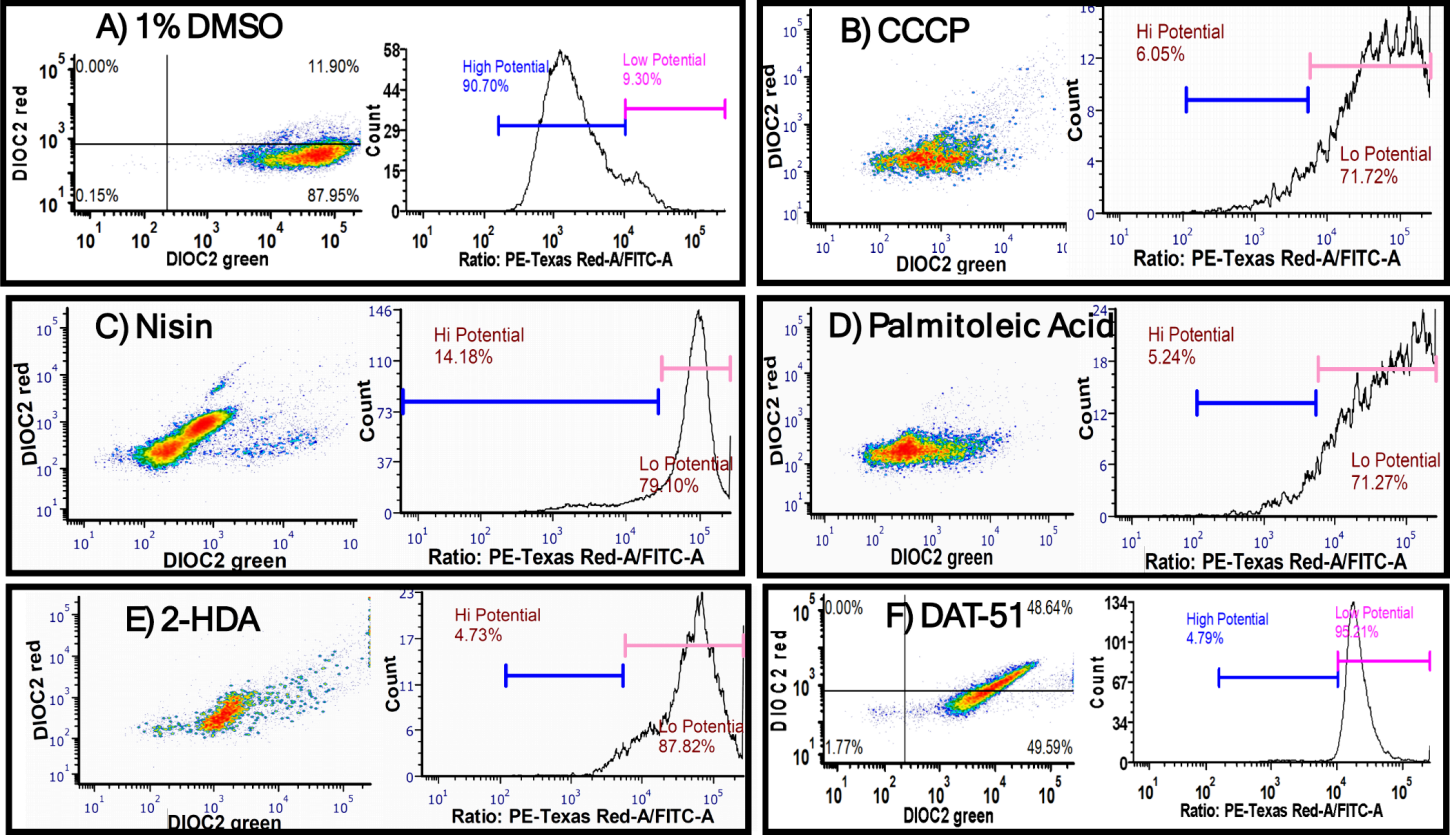
Flow cytometry analysis reveals membrane depolarization in MRSA XIII induced by synthetic FA. Representative dot plots and histograms of MRSA XIII stained with DiOC_2_(3) and TO-PRO-3 after 18–20 h of treatment with 1% DMSO (A), CCCP (B), nisin (C), palmitoleic acid (D), 2-HDA (E), or DAT-51 (F). High- and low-membrane-potential populations were defined by red/green fluorescence ratios by using blue and pink gates, respectively. Analyses were conducted using an LSR Fortessa cytometer and the BacLight Membrane Potential Kit (Thermo). The calibration curve correlating CFU/mL and OD_600_ for MRSA XIII, presented in Figure S1 (see Supporting Information section), was used to standardize bacterial density across flow cytometry experiments.

Cells treated with 2-HDA (4.73%, Figure 7E) and DAT-51 (4.79%, Figure 7F) exhibited similar shifts in fluorescence ratios, indicating strong depolarizing effects. Nisin (Figure 7C) and palmitoleic acid (Figure 7D) treatments also depolarized the membrane, reducing the high potential populations to 14.18% and 5.24%, respectively. In contrast, palmitic acid maintained a profile comparable to that of DMSO (90.70%), consistent with minimal impact on membrane potential (data not shown).

### 3.5 SEM Analysis Reveals Membrane Disruption in MRSA after Treatment with Synthetic uFA

Scanning electron microscopy (SEM) analysis revealed profound morphological alterations in MRSA XIII and MRSA 43300 following treatment with synthetic uFA, as illustrated in Figures 8 and 9. In both strains, treatment with 2-HDA and DAT-51 at 4× MIC disrupted the membrane architecture characterized by pore formation, surface deformation, and extracellular bacterial debris. Specifically, MRSA XIII cells treated with 2-HDA exhibited widespread membrane rupture and accumulation of surface debris (Figure 8D), whereas DAT-51 exposure led to prominent roughening and topological distortion of the bacterial surface (Figure 8E). Similar phenotypes were observed in MRSA 43300, with 2-HDA-treated cells showing extensive membrane damage and DAT-51 causing irregular surface features (Figure 9D,E).

**Figure 8:**
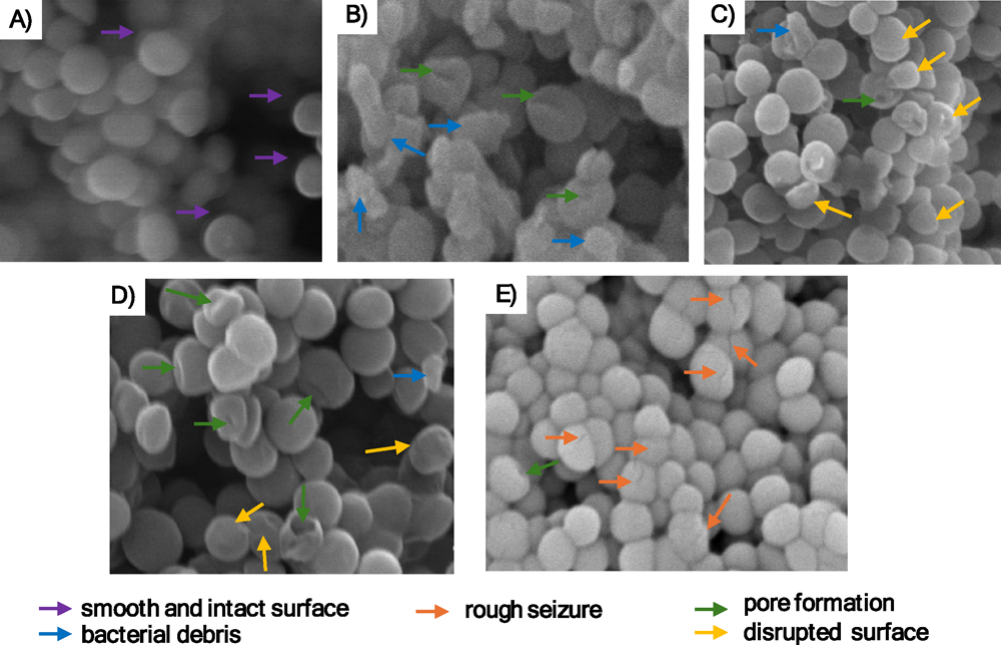
Scanning electron microscopy reveals ultrastructural alterations in MRSA XIII membranes following exposure to synthetic uFA. SEM micrographs illustrate representative morphological changes in MRSA XIII after 18–20 h of treatment with 1% DMSO (A), nisin (B), palmitoleic acid (C), 2-HDA (D), and DAT-51 (E). Images were acquired using a Phenom XL desktop SEM at magnifications ranging from 20,000× to 50,000× and an accelerating voltage of 10 kV. Secondary (SE) and backscattered (BSE) electron detectors were used to capture surface morphology and contrast. In panel (A), purple arrows highlight intact and smooth bacterial surfaces. In contrast, panels (B)–(E) show evidence of membrane damage, including bacterial debris (blue arrows), pore formation (green arrows), rough seizure (orange arrows), and disrupted surfaces (yellow arrows). These panels display selected zoomed-in fields; full SEM images are available in the Supporting Information section (Figures S5–S9).

**Figure 9:**
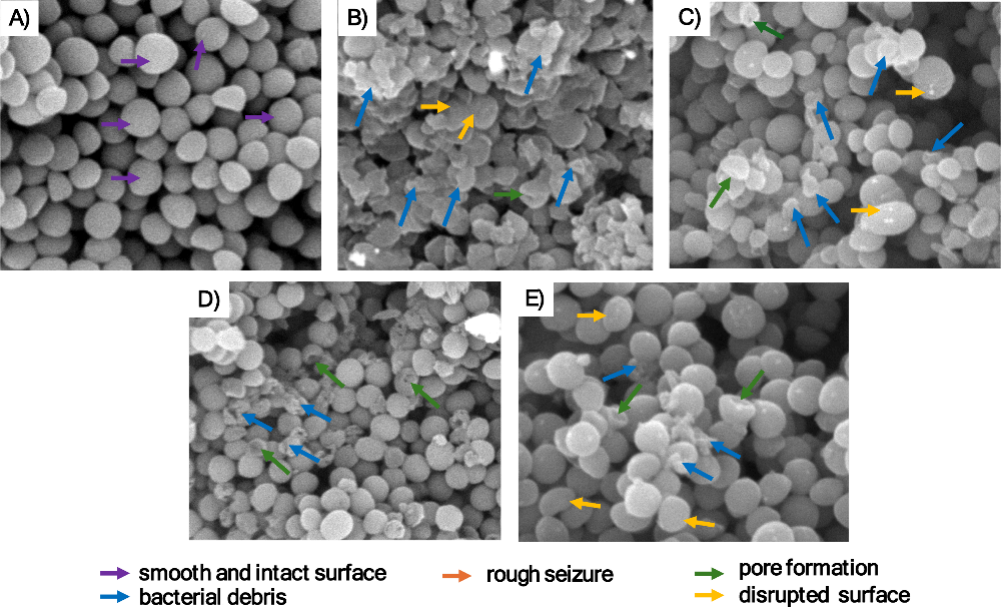
Scanning electron microscopy reveals membrane alterations in MRSA 43300 following treatment with synthetic uFA. Representative SEM micrographs show morphological changes in MRSA 43300 after 18–20 h of exposure to 1% DMSO (A), nisin (B), palmitoleic acid (C), 2-HDA (D), and DAT-51 (E). Images were acquired using a Phenom XL desktop SEM at magnifications ranging from 20,000 to 50,000× and an accelerating voltage of 10 kV. Secondary (SE) and backscattered (BSE) electron detectors were used to capture surface morphology and contrast. Arrows indicate key ultrastructural features: smooth and intact membranes (purple), bacterial debris (blue), pore formation (green), rough membrane surface (orange), and disrupted surfaces (yellow). These panels display selected zoomed-in fields; full SEM images are available in the Supporting Information section (Figures S10–S14).

In contrast, cells exposed to the vehicle control (1% DMSO) retained smooth and intact membranes (Figures 8A and 9A), supporting its role as a negative control. Nisin-treated cells, included as a positive control, exhibited clear evidence of membrane disintegration consistent with its pore-forming activity (Figures 8B and 9B). Cells treated with palmitoleic acid also showed moderate surface disruption and pore formation (Figures 8C and 9C), however, to a reduced extent than those treated with synthetic uFA.

### 3.6 Detection of Lipid II Accumulation, a Key Peptidoglycan Precursor, in *S. aureus* Treated with Synthetic uFA

Lipid II is an essential peptidoglycan precursor that anchors to the inner membrane and serves as a carrier for cell wall subunits during translocation and incorporation into the bacterial cell wall.^66^ Its proper utilization is crucial for maintaining cell wall integrity; thus, its accumulation indicates a blockade in peptidoglycan biosynthesis, leading to impaired cell wall assembly and bacterial stress or death.^52,67^ To assess whether synthetic uFA promotes Lipid II accumulation in MRSA XIII, cells were treated with DAT-51 or 2-HDA at 4× MIC, or with 1% DMSO as vehicle control. Following treatment, cells were lysed, and Lipid II was isolated and biotinylated using PBP4-mediated transpeptidase labeling in the presence of biotinylated d-lysine.^52^ Biotinylated samples were separated by thin-layer chromatography (TLC) and visualized using streptavidin-HRP staining.

As shown in Figure 10, a more intense and distinct band corresponding to biotinylated Lipid II was detected in the DAT-51-treated samples.

**Figure 10:**
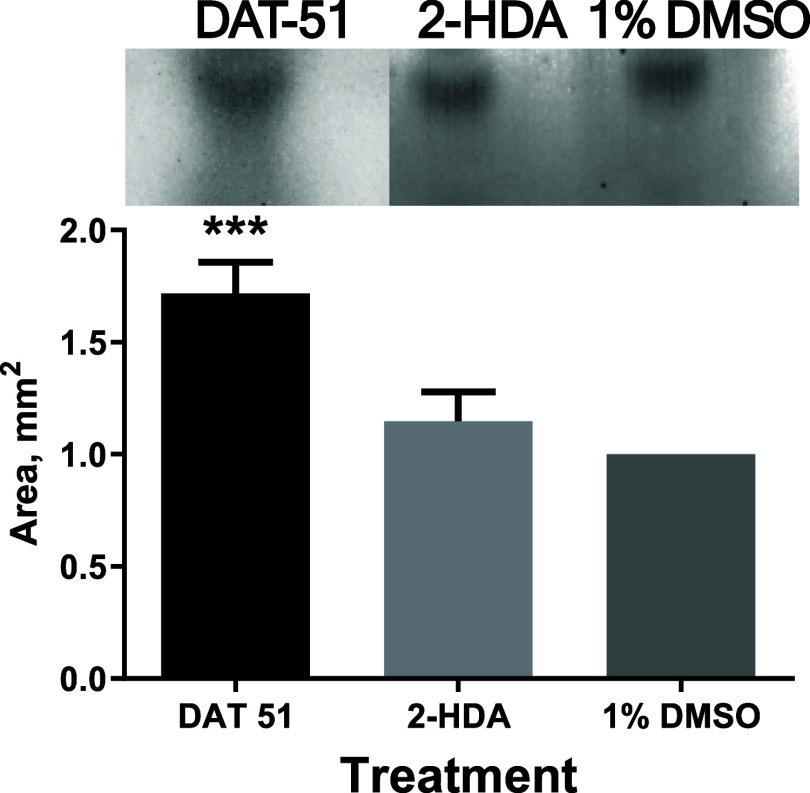
DAT-51 induces lipid II accumulation in MRSA XIII. Densitometric analysis of biotinylated Lipid II in MRSA XIII following treatment with 2-HDA, DAT-51 (4× MIC), or 1% DMSO. TLC was used to resolve Lipid II, and spot areas were quantified with ImageJ. Results are shown as mean ± SEM (*N* = 6).

Densitometric analysis using ImageJ revealed that the average band area for DAT-51 (∼1.72 mm^2^ ± SEM) was significantly greater than that of 2-HDA (∼1.15 mm^2^ ± SEM) and the DMSO control (∼1.00 mm^2^ ± SEM), based on six independent biological replicates (*N* = 6). Statistical analysis using Dunnett’s post hoc test indicated that only DAT-51 induced a significant increase in Lipid II accumulation relative to the control (***p = 0.0007), while no significant difference was observed for 2-HDA (*p* = 0.4552).

### 3.7 Synthetic uFA Inhibit MurA Activity, Blocking the First Step in Peptidoglycan Biosynthesis

To evaluate whether synthetic uFA influences MurA activity, recombinant *S. aureus* MurA was incubated with 2-HDA, DAT-51, linoleic acid, palmitoleic acid, or phosphomycin (positive control) at 4× MIC, and inorganic phosphate release was quantified using a malachite green-based assay. Figure 11 shows that DAT-51, linoleic acid, and phosphomycin treatments reduced enzyme activity relative to the 1% DMSO control.

**Figure 11:**
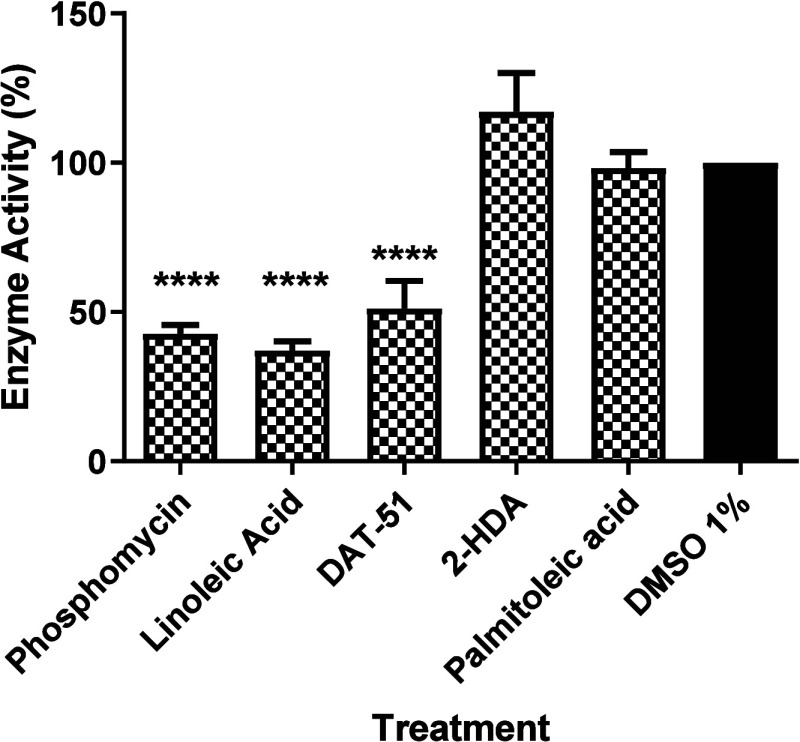
DAT-51 selectively inhibits MurA enzymatic activity in *S. aureus*. MurA activity was evaluated using a malachite green-based colorimetric assay following treatment with 2-HDA, DAT-51, or controls (linoleic acid, palmitoleic acid, phosphomycin, and 1% DMSO vehicle). Reaction mixtures were incubated for 30 min at room temperature after addition of the malachite green working reagent, and absorbance was measured at 620 nm. All reactions were performed in six independent biological replicates, and values are presented as mean ± SEM. One-way ANOVA followed by Dunnett’s multiple comparison test revealed that DAT-51, linoleic acid, and phosphomycin significantly reduced MurA activity compared to the DMSO control (adjusted *p* < 0.0001). In contrast, 2-HDA and palmitoleic acid showed no significant effect on enzyme activity. The MIC values for phosphomycin used in the MurA inhibition assays were determined based on bacterial proliferation experiments using the MTT assay. The complete graph bars and MIC determination for MRSA XIII and the corresponding controls (palmitoleic acid, linoleic acid, and phosphomycin are provided in the Supporting Information (Figures S4, S15, and S16)).

No significant differences were observed following treatment with 2-HDA or palmitoleic acid. The analysis was based on six independent biological replicates, and statistical comparisons were performed using One-Way ANOVA followed by Dunnett’s multiple comparison test.

### 3.8 Comparative Docking Analysis and Sequence Alignment of MurA Enzymes

Molecular docking simulations were conducted using SwissDock to evaluate the binding affinity of five ligands, DAT-51, 2-HDA, linoleic acid, palmitoleic acid, and phosphomycin, toward the *E. coli* MurA (PDB ID: 3KR6). Docking was performed using default parameters, and the best binding modes were selected based on SwissDock scores,^60,61^ and 3D images were generated using the Maestro visualization platform (Schrödinger, LLC, Figure 12). The calculated scores were as follows: DAT-51 (−7.49 kcal/mol), linoleic acid (−7.05 kcal/mol), 2-HDA (−6.88 kcal/mol), palmitoleic acid (−6.75 kcal/mol), and phosphomycin (−5.76 kcal/mol).

**Figure 12:**
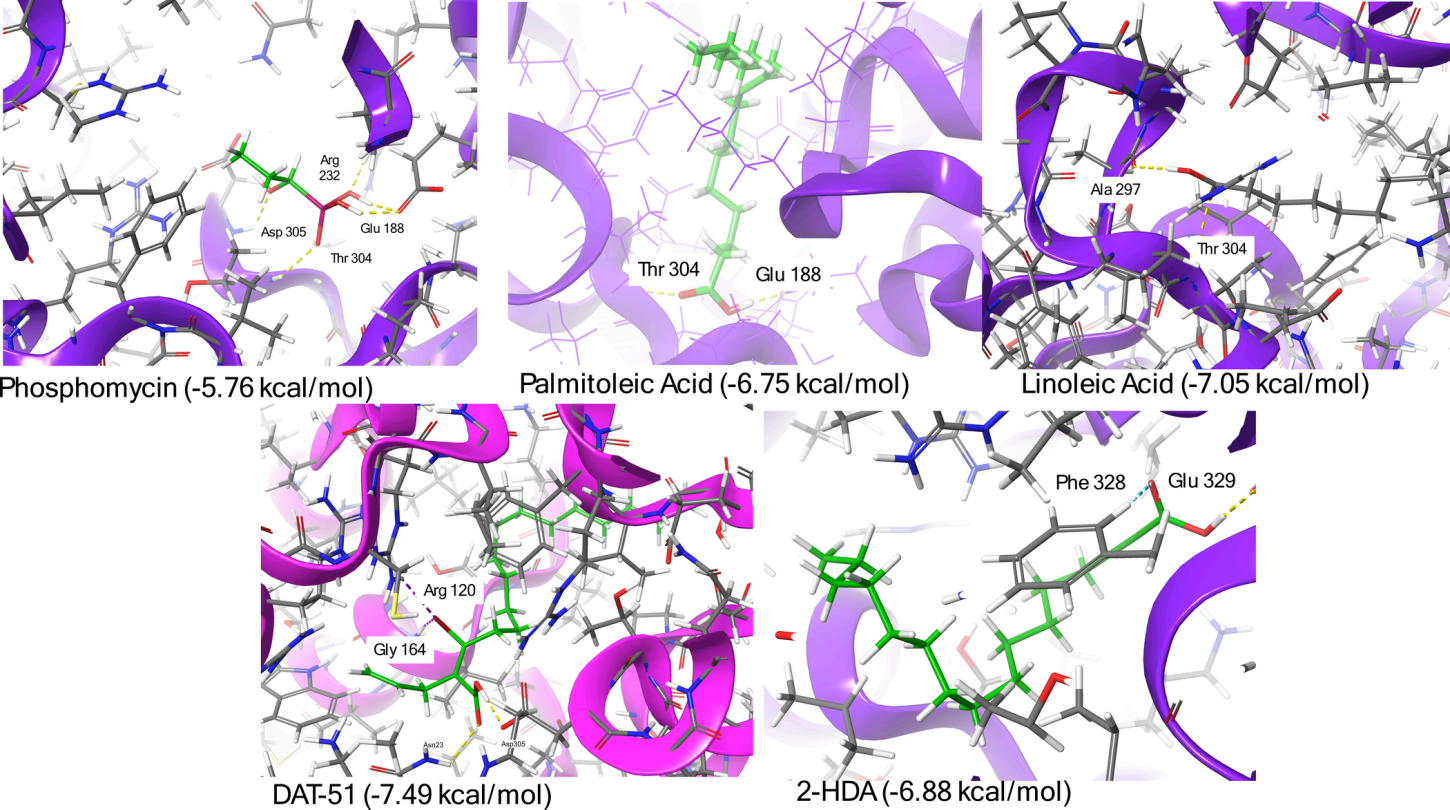
Molecular docking of fatty acids and phosphomycin to *E. coli* MurA (PDB: 3KR6). Molecular docking using SwissDock showing the binding of DAT-51, 2-HDA, linoleic acid, palmitoleic acid, and phosphomycin to the active site of MurA. DAT-51 interacted with Arg 120 & Gly 164. 2-HDA formed noncovalent interactions with Phe 328 & Glu 329. Linoleic and palmitoleic acids interacted near Ala 297 & Thr 304 and Thr 304 & Glu 188, respectively. Phosphomycin showed interactions via Arg 232, Asp 305, Thr 304, and Glu 188. Residues involved are located within the PEP-binding region of MurA.

Among the ligands tested, DAT-51 demonstrated a favorable binding pose within the phosphoenolpyruvate (PEP)-binding pocket of MurA, with a docking score of −7.49 kcal/mol. The molecule was positioned near Arg120 and Gly164, residues implicated in substrate coordination. The orientation of DAT-51 suggested stabilizing noncovalent interactions with these residues, with the bromine atom located near polar side chains within the active site. These interactions may contribute to the observed binding affinity and the proposed inhibitory effect of DAT-51 on MurA.

In contrast, 2-HDA interacted with Phe328 and Glu329, which are located on the periphery of the active site, suggesting weaker or more peripheral binding. Linoleic and palmitoleic acids formed contacts near Ala297, Thr304, and Glu188, residues near the PEP site but without the same anchoring potential as DAT-51. Phosphomycin, used as a positive control, exhibited the expected interactions with key catalytic residues, including Arg232, Asp30, Thr304, and Glu188, thereby corroborating the validity of the docking setup.

To assess the structural relevance of the *E. coli* MurA model for use in *S. aureus*, we conducted a pairwise sequence alignment between the MurA protein from *E. coli* (PDB 3KR6) and the MurA sequence from *S. aureus*, obtained from UniProt (ID: Q2FWD4). The alignment included 419 amino acids, and we recorded 100% sequence identity and alignment metrics to confirm its suitability for comparative modeling.

### 3.9 Synthetic uFAs Alter the Composition of Phospholipid and Free Fatty Acid Fractions in MRSA XIII

To evaluate the impact of synthetic uFA on the endogenous FA profile of MRSA XIII, GC-MS analysis was performed on lipid extracts fractionated into phospholipid and free FA pools following treatment with 2-HDA or DAT-51 at 4× MIC as described in the literature.^32,45^ As illustrated in Figure 13, 2-HDA was detected in both lipid fractions, whereas DAT-51 was not detected in any of the four biological replicates analyzed by GC-MS analysis, indicating an absence of measurable incorporation into either lipid fraction under the tested conditions.

**Figure 13:**
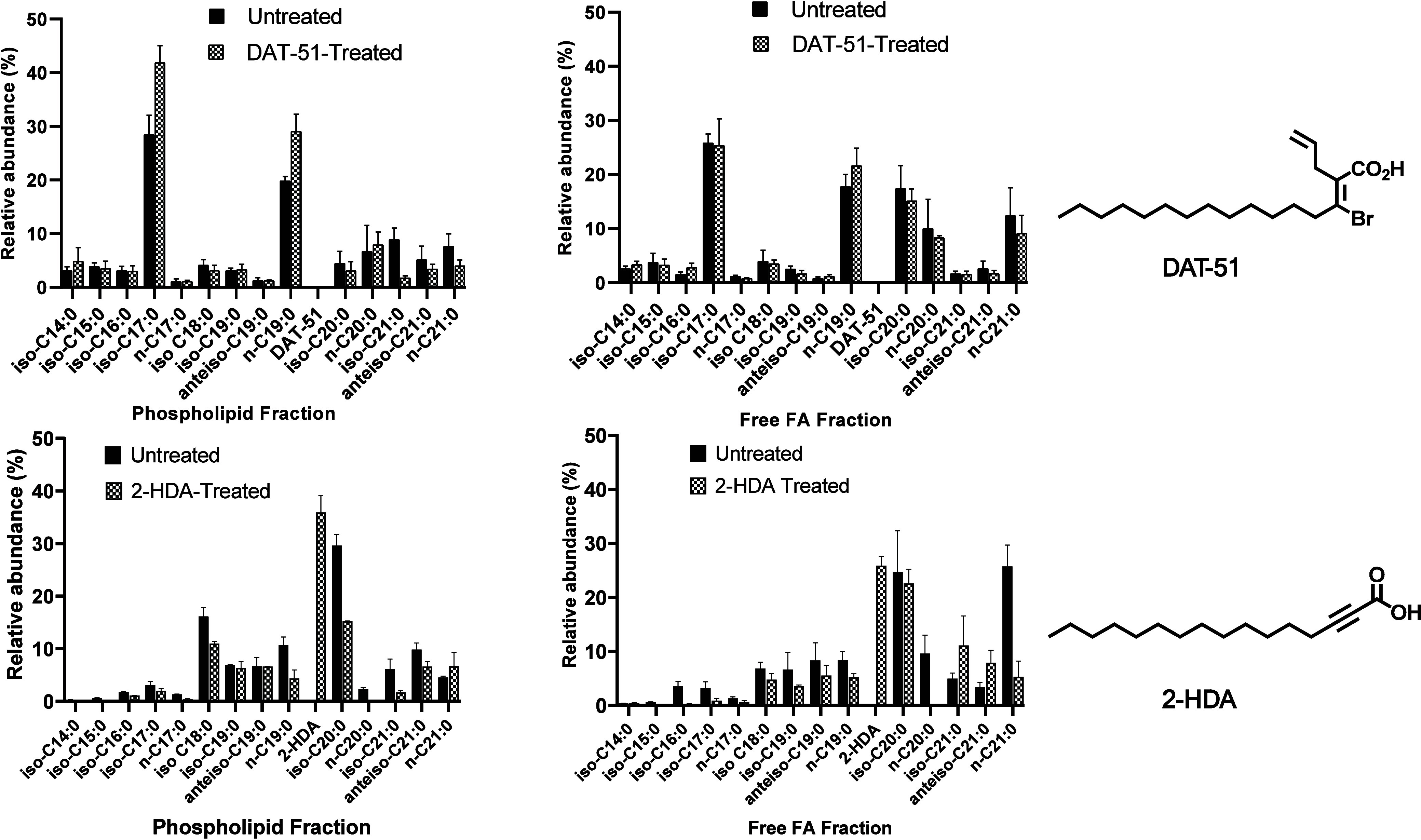
Synthetic uFAs alter FA composition in MRSA XIII. A GC-MS analysis was performed to assess changes in the endogenous FA profile of MRSA XIII following exposure to synthetic uFA. Bacterial cultures were treated with 2-HDA or DAT-51 at 4× MIC and incubated for 18–20 h at 37 °C. Post-treatment, lipid extracts were fractionated into phospholipid and free FA pools, derivatized to fatty acid methyl esters (FAMEs), and analyzed by GC-MS. The graphs depict the relative abundance (%) of bacterial FA in untreated (black bars) versus uFA-treated (patterned bars) samples.

Treatment with 2-HDA led to quantifiable incorporation into the phospholipid and free FA pools with relative abundances distinguishable from untreated controls. In contrast, the DAT-51-treated samples showed no chromatographic evidence of compound retention. Additionally, the relative distribution of endogenous bacterial FA, such as iso-C17:0, anteiso-C17:0, and iso-C19:0, varied depending on the treatment condition.

### 3.10 Cytotoxicity Assessment of 2-HDA in Vero Cells Using the MTT Assay

In Vero cells (ATCC CCL-81), 2-HDA produced a dose-dependent decrease in MTT signal after 18–20 h, with statistically significant reductions at 250–1000 μg/mL (****p < 0.0001 vs 1% DMSO untreated control); lower concentrations were not different from control (Figure S17). By contrast, we recently reported that DAT-51 exhibited minimal cytotoxicity in the MTS assay, showing ∼19% reduction only at 100 μg/mL,^33^ consistent with our findings with 2-HDA.

### 3.11 2-HDA Modulates Biophysical Properties of DOPC Model Membranes

To examine the membrane-specific effects of 2-HDA, we evaluated its impact on the biophysical properties of DOPC model membranes.^68^ Lipids are abundant in mammalian cell membranes.^69^ Given 2-HDA’s low cytotoxicity in Vero cells, DOPC bilayers were used to assess changes in key biophysical parameters, including osmotic water permeability (membrane barrier function), phase transition temperature and enthalpy (thermal behavior and lipid packing), and hydrocarbon chain order (membrane fluidity and organization), upon incorporating 2-HDA (Figure 14).

**Figure 14:**
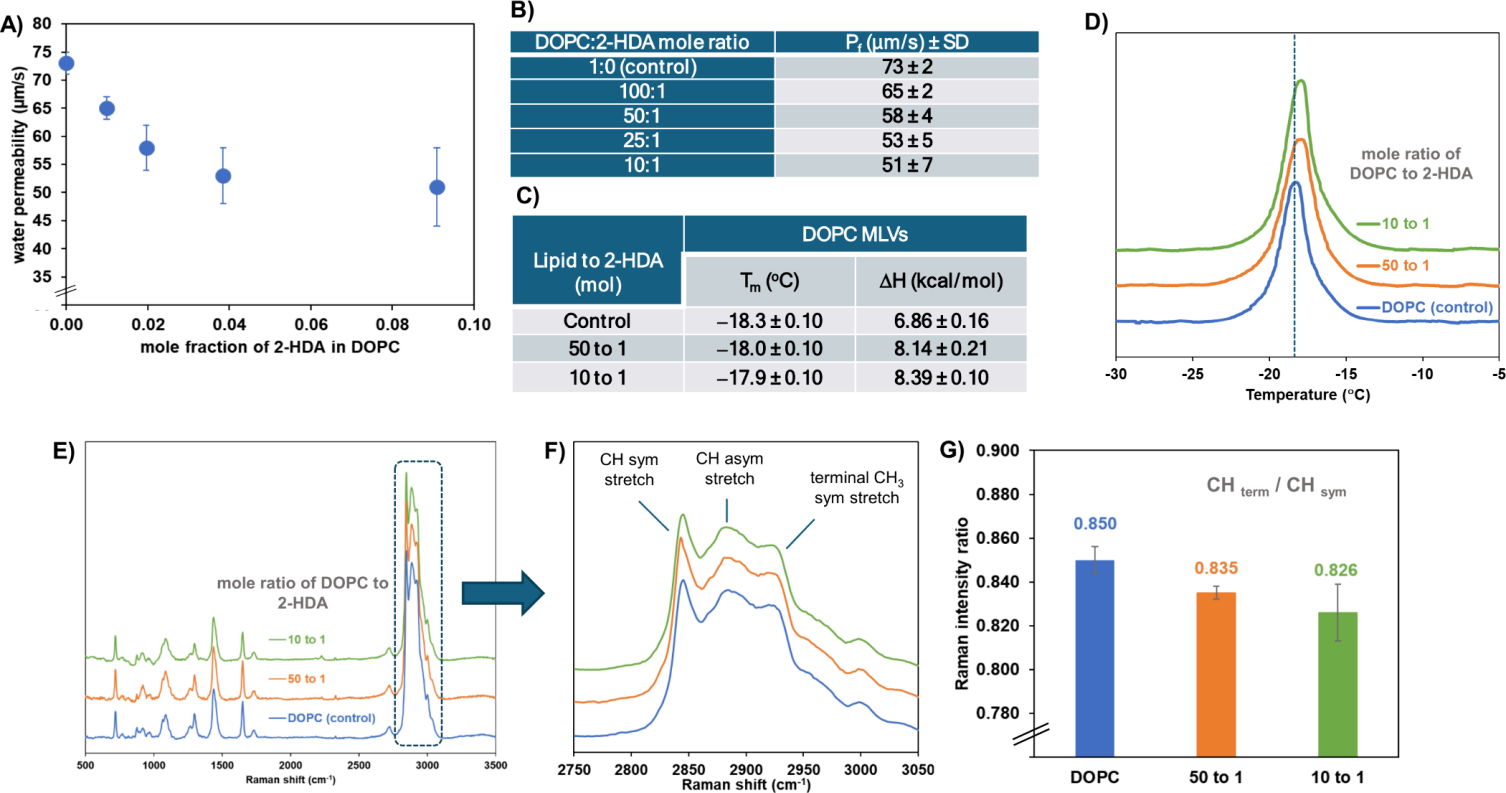
Concentration-dependent biophysical effects of 2-HDA on the DOPC model membranes. (A) Osmotic water permeability (Pf) of DOPC bilayers at 30 °C as a function of 2-HDA mole fraction. (B) Tabulated Pf values (μm/s ± SD) for the DOPC:2-HDA molar ratios. (C) Thermodynamic parameters (*T*_m_ and Δ*H*) derived from differential scanning calorimetry (DSC) of DOPC MLVs with increasing 2-HDA concentrations. (D) DSC thermograms showing endothermic transitions of DOPC MLVs; the dotted line indicates *T*_m_ of the control. (E) Raman spectra (500–3500 cm^–1^) of supported DOPC bilayers with or without 2-HDA. (F) CH stretching region (2750–3050 cm^–1^) highlighting CH_2_ symmetric/asymmetric and CH_3_ terminal stretches. (G) Raman intensity ratio [CHterm/CHsym] as a function of the 2-HDA concentration. Spectra in panels (E) and (F) are vertically offset for the sake of clarity.

Osmotic swelling measurements showed a concentration-dependent decrease in water permeability (Pf), from 73 ± 2 μm/s in pure DOPC to 51 ± 7 μm/s in a DOPC:2-HDA ratio of 10:1 (Figure 14B). DSC thermograms revealed a shift in the phase transition temperature (*T*_m_) from −18.3 °C in the control (consistent with the literature value)^70^ to −18.0 °C and −17.9 °C at 50:1 and 10:1 ratios, respectively, with corresponding increases in enthalpy (Δ*H*) to 8.14 and 8.39 kcal/mol (Figure 14C,D).

Raman microscopy was used to examine how 2-HDA affects the structural organization of supported DOPC bilayers. As shown in Figure 14E,F, the CH stretching region (2750–3050 cm^–1^) revealed characteristic peaks at ∼2845 cm^–1^ (CH_2_ symmetric), ∼2890 cm^–1^ (CH_2_ antisymmetric), and ∼2930 cm^–1^ (CH_3_ symmetric).^71,72^ The ratio of terminal methyl to methylene symmetric stretch intensities ([CHterm/CHsym]) decreased from 0.850 (control) to 0.826 (10:1 2-HDA) (Figure 14G), indicating reduced chain mobility and increased bilayer order.^73^ These spectral changes support a stabilizing effect of 2-HDA on the membrane packing.

## 4 Discussion

The increasing prevalence of antibiotic-resistant bacterial infections, particularly those caused by MRSA, underscores the urgency of developing novel antibacterial agents.^74–76^ In this study, we evaluated the mechanisms of action of synthetic uFA 2-HDA and DAT-51 (Figure 1) against MRSA XIII or MRSA ATCC 43300, integrating membrane integrity assays, enzymatic inhibition studies, lipidomic profiling, and cytotoxicity assessment in eukaryotic cells. The convergence of these methodologies offers a multifaceted understanding of these compounds’ antibacterial potential and selectivity.

Our initial interest in 2-HDA and DAT-51 stems from extensive prior work on synthetic uFA.^27,32,33^ In a pivotal study by our group, a series of acetylenic FA were synthesized and evaluated for antibacterial activity, leading to the identification of 2-HDA as the most active compound against *S. aureus*, including methicillin-resistant strains.^27^ Building upon this foundation, we later synthesized various isomers of 2-HDA, as well as sulfur-substituted analogs, and consistently found that 2-HDA maintained superior potency.^32^ More recently, we developed DAT-51, a halogenated allyl derivative of 2-HDA, which demonstrated even greater activity against MRSA and was shown to induce significant fluorescein permeabilization in treated cells.^33^ This observation strongly suggested that the bacterial plasma membrane is a critical target for these compounds. Our hypothesis is further supported by the findings of Parsons and collaborators, who demonstrated that certain naturally occurring uFAs, such as palmitoleic acid, provoke cytoplasmatic membrane disruption and cause leakage of low-molecular-weight proteins in *S. aureus*.^22^ Altogether, these prior studies establish a clear rationale for selecting 2-HDA and DAT-51 as lead compounds and investigating membrane disruption as a key mechanism underlying their antibacterial activity.

To evaluate the antibacterial efficacy of 2-HDA and DAT-51, we first assessed their ability to inhibit the proliferation of MRSA XIII and MRSA ATCC 43300 using the MTT assay (Figure 2). Both compounds exhibited concentration-dependent antibacterial activity, with 2-HDA demonstrating greater potency at lower concentrations. Building on these results, we explored whether this inhibitory effect could be attributed to membrane-targeting mechanisms. The two independent assays, qualitative DAPI/PI microscopy (Figures 3 and 4) and quantitative TO-PRO-3 iodine kinetics (Figure 5), converge on the same outcome: 2-HDA and DAT-51 compromise MRSA membrane integrity. The kinetic step-then-plateau profiles indicate a rapid loss of barrier function followed by equilibration of dye entry. Meanwhile, the DAPI/PI fields neatly separate vehicles from nisin and uFA-treated cells across both strains. Attenuation at the highest dose (4× MIC), especially for 2-HDA, is consistent with self-association above the critical micelle concentration (CMC), which depletes the membrane-insertable monomer pool and can dampen far-red fluorescence via aggregation-caused quenching. Our group has previously reported this micellization behavior for 2-HDA.^27^ Strain-dependent differences (larger amplitudes in MRSA XIII than MRSA ATCC 43300, and a shifted dose rank in MRSA ATCC 43300) are expected from variations in cell-envelope composition and surface charge that modulate amphiphile insertion and dye uptake, consistent with the framework established by Xu et al. for impermeant-dye and depolarization readouts.^50^

The inclusion of MRSA ATCC 43300 in this study is especially relevant, as this strain is widely used as a standard control in antimicrobial susceptibility testing for methicillin-resistant *S. aureus* due to its well-characterized resistance profile and reproducible phenotypic behavior.^77–79^ The parallel observation of membrane permeabilization in both the clinical isolate (MRSA XIII) and the reference strain (MRSA 43300) further supports the generalizability of these findings. Consistent with this, DNA/RNA leakage assays (Figure 6) demonstrated that 2-HDA induced the most pronounced and time-dependent release of intracellular nucleic acids, further supporting the hypothesis that synthetic uFA compromises bacterial membrane integrity.

Leakage was observed as early as the initial time point following exposure to 2-HDA, DAT-51, and nisin, as indicated by the elevated absorbance at 260 nm (see Figure 6). This fast release of intracellular genetic material suggests that membrane disruption by these agents occurs very fast upon contact with the bacterial envelope. While this phenomenon may initially appear surprising, it is increasingly supported by studies on membrane-active compounds. For example, Yasir et al. demonstrated that the cationic antimicrobial peptide melimine triggered a significant release of DNA/RNA from *Pseudomonas aeruginosa* within just 2 min of exposure, highlighting the capacity of specific agents to permeabilize bacterial membranes rapidly and induce leakage of large cytoplasmic molecules.^80^ Therefore, our findings, showing immediate leakage upon treatment with synthetic uFA, represent a novel observation in the context of FA-based antimicrobials and underscore the potent and rapid membrane-disrupting action of 2-HDA and DAT-51, especially at 4xMIC concentrations.

Flow cytometry analysis using DiOC_2_(3) staining (Figure 7) provided additional mechanistic insight by revealing significant membrane depolarization in MRSA XIII cells treated with 2-HDA and DAT-51. The observed depolarization levels were comparable to those induced by known membrane-active compounds such as CCCP and nisin, indicating the disruption of electrochemical gradients essential for bacterial viability. These results are consistent with those obtained by fluorescence microscopy (Figures 3 and 4), where DAPI/PI live-dead microscopy membrane-permeability assays showed compromised membrane permeability, and with the immediate release of nucleic acids observed in the DNA/RNA leakage assay (Figure 6). The analysis of evidence across three independent methodologies, membrane permeabilization (Figures 3–5), leakage of intracellular contents (Figure 6), and membrane potential disruption (Figure 7), provides robust and internally consistent validation of the rapid membrane-disrupting activity of 2-HDA and DAT-51.

Similar effects have been reported with other uFAs. For instance, palmitoleic acid has been shown to induce rapid membrane depolarization and leakage of intracellular contents in *S. aureus*, leading to growth inhibition.^22^ Additionally, studies employing flow cytometry have demonstrated that exposure to FA results in significant membrane depolarization in various bacterial species, further corroborating the membrane-disruptive properties of this class of compounds.^81^

Scanning electron microscopy (Figures 8 and 9) confirmed the structural consequences of these disruptions. MRSA strains treated with 2-HDA and DAT-51 showed marked surface damage, pore formation, and cellular debris, corroborating biochemical indicators of membrane compromise. Particularly, the severity of structural changes reflected trends observed in both flow cytometry and leakage assays, validating the impact of these FA at multiple levels of membrane analysis. Similar structural alterations have been reported in MRSA cells treated with other membrane-active agents. SEM revealed pore formation, membrane collapse, and surface irregularities consistent with membrane disruption mechanisms confirmed by complementary biochemical and flow cytometry assays.^82,83^

Peptidoglycan biosynthesis is a vital bacterial process reliant on early cytoplasmic steps catalyzed by MurA and culminating in the production of Lipid II, a membrane-bound precursor and key antimicrobial target.^84,85^ Despite the clinical importance of these targets, to the best of our knowledge, no prior studies have explored the simultaneous impact of synthetic uFA on both MurA activity and Lipid II accumulation. In this context, we evaluated the ability of DAT-51 and 2-HDA to interfere with peptidoglycan biosynthesis, membrane stability, and Lipid II dynamics in MRSA. TLC-based analysis revealed that DAT-51 treatment leads to significant accumulation of Lipid II (Figure 10), suggesting that it interferes with early biosynthetic steps. This was further supported by the observed decrease in MurA activity (Figure 11). This effect was not observed with 2-HDA, despite its potent antibacterial activity. One plausible explanation lies in the differential impact of each compound on membrane integrity. Based on live/dead experiments, nucleic acid leakage, and SEM assays (Figures 3–9), 2-HDA exhibits a more pronounced pore-forming activity than DAT-51, likely resulting in the extensive loss of cytoplasmic content and degradation or dispersion of lipid intermediates, such as Lipid II. In contrast, DAT-51 appears to moderately disrupt the membrane while preserving sufficient intracellular organization to accumulate biosynthetic intermediates, a scenario compatible with partial or indirect MurA inhibition. This interpretation is supported by studies of pore-forming antimicrobials like nisin, which binds Lipid II with high affinity and sequesters it as part of its pore-forming mechanism, disrupting its role in cell wall synthesis and potentially reducing its detectable accumulation.^86^ Furthermore, Parsons et al. reported that the degree of membrane permeabilization caused by uFA is associated with selective loss of small molecules and structural damage in *S. aureus*.^22^ Collectively, these findings support a model in which DAT-51 exerts a dual mechanism of action, moderate membrane disruption that enables access to cytoplasmic targets, combined with interference in early steps of peptidoglycan biosynthesis, whereas 2-HDA acts primarily through aggressive membrane destabilization.

The divergent physicochemical properties of 2-HDA and DAT-51 probably contribute to their distinct biological effects. Mainly, DAT-51 exhibits a higher calculated partition coefficient (cLogP = 8.454) compared to 2-HDA (cLogP = 6.504), indicating a stronger hydrophobic character and a greater propensity to associate with membrane interfaces rather than integrate deeply into the lipid bilayer.^87^ This may explain DAT-51’s moderate membrane-disrupting activity and its ability to accumulate intracellularly and engage MurA, as opposed to 2-HDA, which has a relatively lower cLogP, favoring greater membrane insertion, resulting in pore formation and the rapid efflux or degradation of lipid intermediates like Lipid II. These observations align with the idea that indirect changes in lipophilicity can significantly influence the mechanism of action of membrane-active antimicrobials.

The malachite green-based colorimetric assay (Figure 11) demonstrated that DAT-51 significantly inhibited *S. aureus* MurA activity compared to the DMSO control (*p* < 0.0001), with inhibition levels comparable to those of linoleic acid and the covalent MurA inhibitor phosphomycin. In contrast, 2-HDA and palmitoleic acid showed no significant inhibition under identical conditions.

Results displayed in Figure 11 align with molecular docking data from Figure 12, where DAT-51 showed the strongest predicted binding to MurA (−7.50 kcal/mol), followed by binding to linoleic acid, 2-HDA, and palmitoleic acid. Despite its known potency, phosphomycin yielded lower scores due to its covalent binding mechanism,^88^ consistent with previous reports.^89^ These trends support the *in vitro* inhibition observed for DAT-51 and related fatty acids.

As further support, it was reported that lauric acid, a natural saturated fatty acid structurally similar to our synthetic uFAs, also displayed stronger MurA binding than phosphomycin.^89^ This reinforces the concept that fatty acid scaffolds can inhibit the early steps of peptidoglycan biosynthesis.

Lipidomic profiling through GC-MS (Figure 13) provided further mechanistic insight into compound-bacteria interactions at the membrane level. 2-HDA was detected in MRSA’s phospholipid and free fatty acid fractions, suggesting direct incorporation into the bacterial envelope. This observation is consistent with previous findings from our group, where 2-HDA was also detected in membrane-associated lipid fractions in *S. aureus* and *Escherichia coli*, supporting its ability to integrate into bacterial membranes and perturb their structural organization.^32,45^ Such incorporation probably contributes to 2-HDA’s pronounced membrane-targeting activity, as reflected across permeability (Figures 3–5), nucleic acid leakage assays (Figure 6), and membrane potential (Figure 7). It may also explain the absence of detectable Lipid II accumulation in 2-HDA-treated cells. Extensive membrane disruption caused by 2-HDA could result in the degradation or efflux of lipid intermediates, consistent with findings reported for other pore-forming agents such as nisin.^86^ In contrast, DAT-51 was not detected in either lipid fraction, suggesting that its membrane effects are likely due to peripheral interactions rather than full incorporation. This aligns with its intracellular activity targeting MurA and promoting Lipid II accumulation (Figure 10), made possible by moderate membrane permeabilization, which preserves the cytoplasmic machinery necessary for precursor buildup.

Also, in Figure 13, we observed variations in the abundance of specific FA, particularly *iso*-C17:0 and *n*-C19:0, among untreated control samples (1% DMSO) across biological replicates. In untreated samples, these discrepancies likely reflect intrinsic physiological variability in MRSA XIII, consistent with previous reports showing that *S. aureus* modulates its membrane FA composition in response to growth phase, nutrient availability, and metabolic state.^90^ Such fluctuations are especially evident in branched-chain fatty acids derived from amino acid catabolism (e.g., leucine and isoleucine), which are known to vary in response to cellular stress and population density.^90–92^

Cytotoxicity assays in Vero cells (Figure S17) confirmed that 2-HDA did not show cytotoxicity below 250 μg/mL. At higher concentrations, proliferation decreased significantly, although bacterial MICs are much lower, suggesting a favorable therapeutic window. Additionally, we previously reported that DAT-51 exhibits low cytotoxicity in Vero cells, with only a ∼19% reduction in viability at 100 μg/mL, further supporting the therapeutic potential of these synthetic uFA.^33^

The biophysical analysis of 2-HDA in DOPC membranes model (Figure 14) demonstrates a concentration-dependent reduction in water permeability, increased thermotropic enthalpy, and enhanced acyl chain packing, suggesting that 2-HDA rigidifies model membranes,^68,93–95^ in a manner comparable to palmitic acid. This behavior aligns with its low cytotoxicity in Vero cells, indicating selectivity toward bacteria over mammalian membranes. On the other hand, 2-HDA disrupts bacterial membrane integrity, as evidenced by membrane permeabilization, nucleic acid leakage, depolarization, and its incorporation into phospholipid fractions, without causing Lipid II accumulation, likely due to extensive membrane damage and efflux of intermediates. Indirectly, these findings suggest that membrane destabilization is the primary antibacterial mechanism of 2-HDA. Conversely, DAT-51 exhibits a dual mode of action: it moderately disrupts bacterial membranes, inhibits MurA activity, and induces Lipid II accumulation, consistent with peripheral membrane interactions and intracellular target engagement. Together, these findings support the idea that synthetic uFAs exert antibacterial activity through different concentration- and structure-dependent biophysical mechanisms and highlight the selective action of 2-HDA and DAT-51 against bacterial membranes. This selectivity reinforces their potential as lead scaffolds, consistent with previous observations that bacterial membranes are inherently more susceptible to uFA-induced disruption in comparison to their mammalian counterparts.

## 5 Conclusions

In summary, our findings support a dual-action model for the synthetic uFA. 2-HDA acts predominantly through membrane disruption, consistent with its strong pore-forming activity. On the other hand, DAT-51 exhibits a mixed mechanism involving moderate membrane perturbation coupled with interference in peptidoglycan biosynthesis, probably through MurA enzymatic inhibition and precursor accumulation. These mechanistic differences appear to stem from distinct structural features and modes of membrane interaction. These compounds’ selectivity and complementary antibacterial activities highlight their potential as scaffolds for developing dual-target antimicrobials. Future studies should aim to define the structural determinants of intracellular target action and membrane incorporation and validate the *in vivo* efficacy of these agents against resistant Gram-positive pathogens.

## Supplementary Material



## Notes

Standard curve correlating OD600 with viable cell counts in MRSA XIII (Figure S1); Dose-dependent inhibition of MRSA XIII and MRSA ATCC 43300 proliferation by nisin (Figures S2–S3); Dose-dependent inhibition of MRSA XIII by palmitoleic acid (Figure S4); Scanning electron micrographs (SEM) of MRSA XIII and MRSA ATCC 43300 under different treatments: vehicle control, nisin, palmitoleic acid, 2-HDA, and DAT-51 (Figures S5–S14); Dose-dependent inhibition of MRSA XIII proliferation by linoleic acid (Figure S15); Dose-dependent inhibition of MRSA XIII proliferation by phosphomycin (Figure S16); Cytotoxicity of 2-HDA in Vero cells assessed by MTT assay (Figure S17) (PDF)
