# Supplementary material for: Dual Mechanisms of Action of a Halogenated Allyl Fatty Acid against Methicillin-Resistant Staphylococcus aureus
Source: ACS Omega. 2025 Nov 13;10(46):55769–86. doi: 10.1021/acsomega.5c07162 (PMC12658796; doi:10.1021/acsomega.5c07162)
Supplement: Supplementary file 1 [file ao5c07162_si_001.pdf]

Supporting information:

Dual Mechanisms of Action of a Halogenated Allyl Fatty Acid Against Methicillin-Resistant Staphylococcus Aureus

Jazmar Villarini-Torres[a,b], Giancarlo Casillas-Vargas[a], Karama Shayeb[a,b], Alexis Rosado-Ortíz[a,b], Luzmarie Reyes-Vicente[a,b], Mayerli De Jesús-Vega[a,b], Derik Amely-Gavilán [a,b], Natasha Díaz-Cruz [a,b], Gil Cortés-Rodríguez [c], Jessica Said [d], Jasmin Ceja-Vega [d], Elizabeth Andersen [d], Amani Rabadi [d], Antonio Colom [a], Harry Rivera [c], Sunghee Lee [d], Nataliya Chorna [e], Kathleen Brundage [f], Néstor M. Carballeira [g], David J. Sanabria-Ríos [a,b]

[a]Department of Natural Sciences, Inter American University of Puerto Rico, Metropolitan Campus, P.O. Box 191293, San Juan, Puerto Rico 00919, USA

[b]Medicinal Research and Applications Laboratory, Inter American University of Puerto Rico, Metropolitan Campus, P.O. Box 191293, San Juan, Puerto Rico 00919, USA

[c]Department of Natural Sciences and Mathematics, Inter American University of Puerto Rico, Bayamón Campus, 500 Road Dr., John Will Harris, Bayamón, PR 00957, USA

[d]Department of Chemistry and Biochemistry, Iona University, 715 North Avenue, New Rochelle, New York 10801, USA

[e]Department of Biochemistry, Medical Sciences Campus, University of Puerto Rico, P.O. Box 365067, San Juan, PR 00936, USA

[f]Department of Microbiology, Immunology & Cell Biology, West Virginia University P.O. Box 9177, Morgantown, WV 26506, USA

[g]Department of Chemistry, University of Puerto Rico, Río Piedras Campus, 17 Ave Universidad STE 1701, San Juan, PR 00925, USA

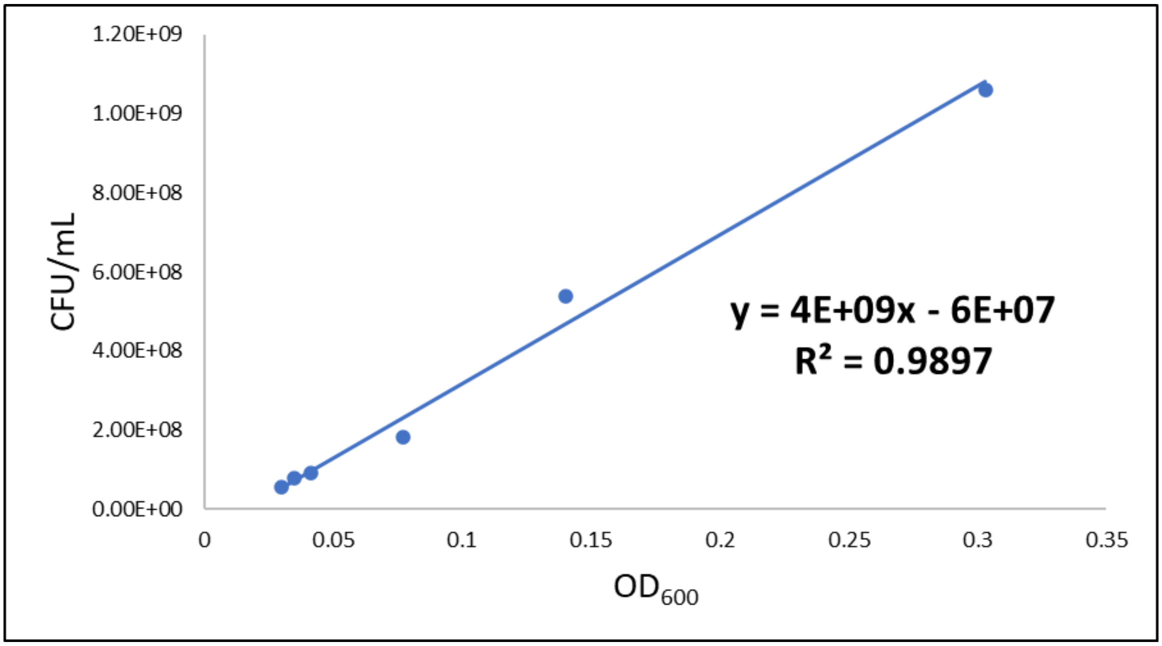

Figure S1. *Standard Curve Correlating OD_{600} with Viable Cell Count in MRSA XIII*. Linear regression analysis of MRSA XIII demonstrating a strong correlation (R^2 = 0.9897) between optical density at 600 nm (OD_{600}) and colony-forming units per milliliter (CFU/mL). This standard curve enables the estimation of viable bacterial concentration from OD_{600} measurements in flow cytometry experiments.

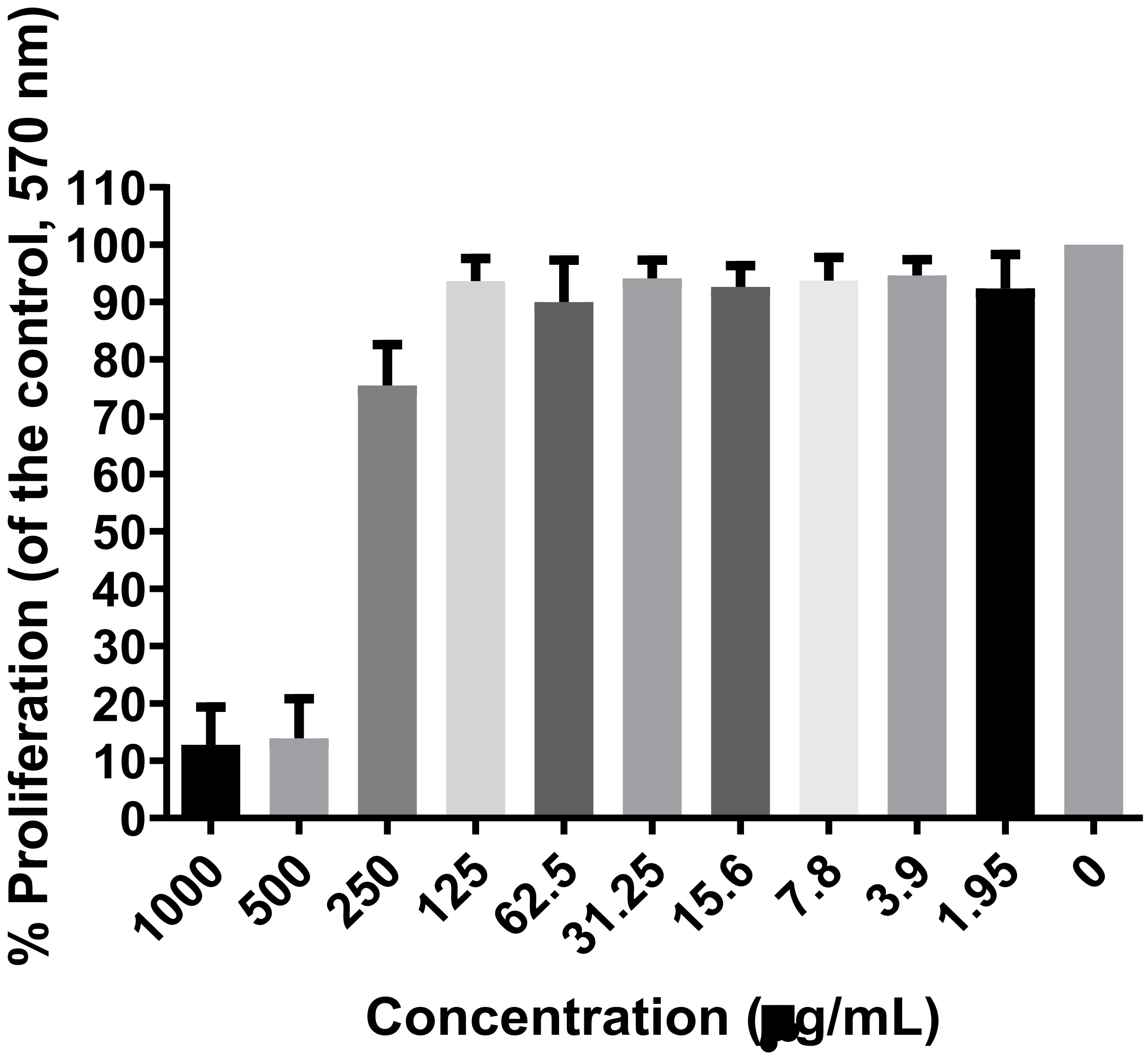

Figure S2. *Dose-Dependent Inhibition of MRSA XIII Proliferation by Nisin.* MRSA XIII cultures were treated with increasing concentrations of nisin for 18-20 hours at 37 °C. Bacterial proliferation was quantified via the MTT assay and expressed as a percentage of the untreated control (0 µg/mL) at 570 nm. Bars represent the mean ± SEM from six independent biological replicates. A marked dose-dependent reduction in viability was observed, with the minimum inhibitory concentration (MIC) determined to be 500 µg/mL under these conditions. This MIC value was subsequently used for the experimental design of biochemical assays.

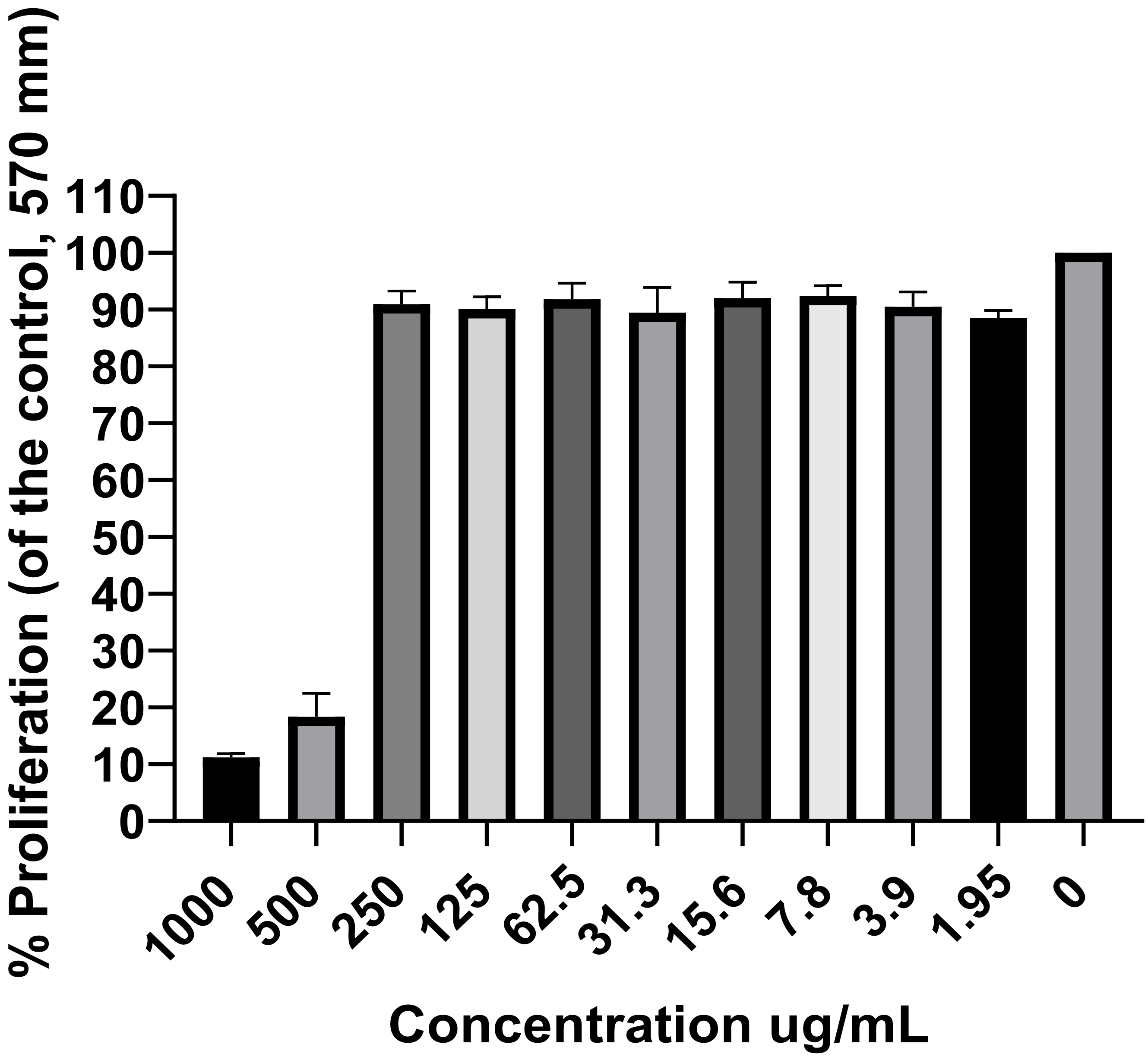

Figure S3. *Dose-Dependent Inhibition of MRSA ATCC 43300 Proliferation by Nisin*. MRSA ATCC 43300 cultures were treated with increasing nisin concentrations for 18-20 hours at 37 °C. Bacterial proliferation was quantified via the MTT assay and expressed as a percentage of the untreated control (0 µg/mL) at 570 nm. Bars represent the mean ± SEM from six independent biological replicates. A marked dose-dependent reduction in viability was observed, with the minimum inhibitory concentration (MIC) determined to be 500 µg/mL under these conditions. This MIC value was subsequently used for the experimental design of biochemical assays.

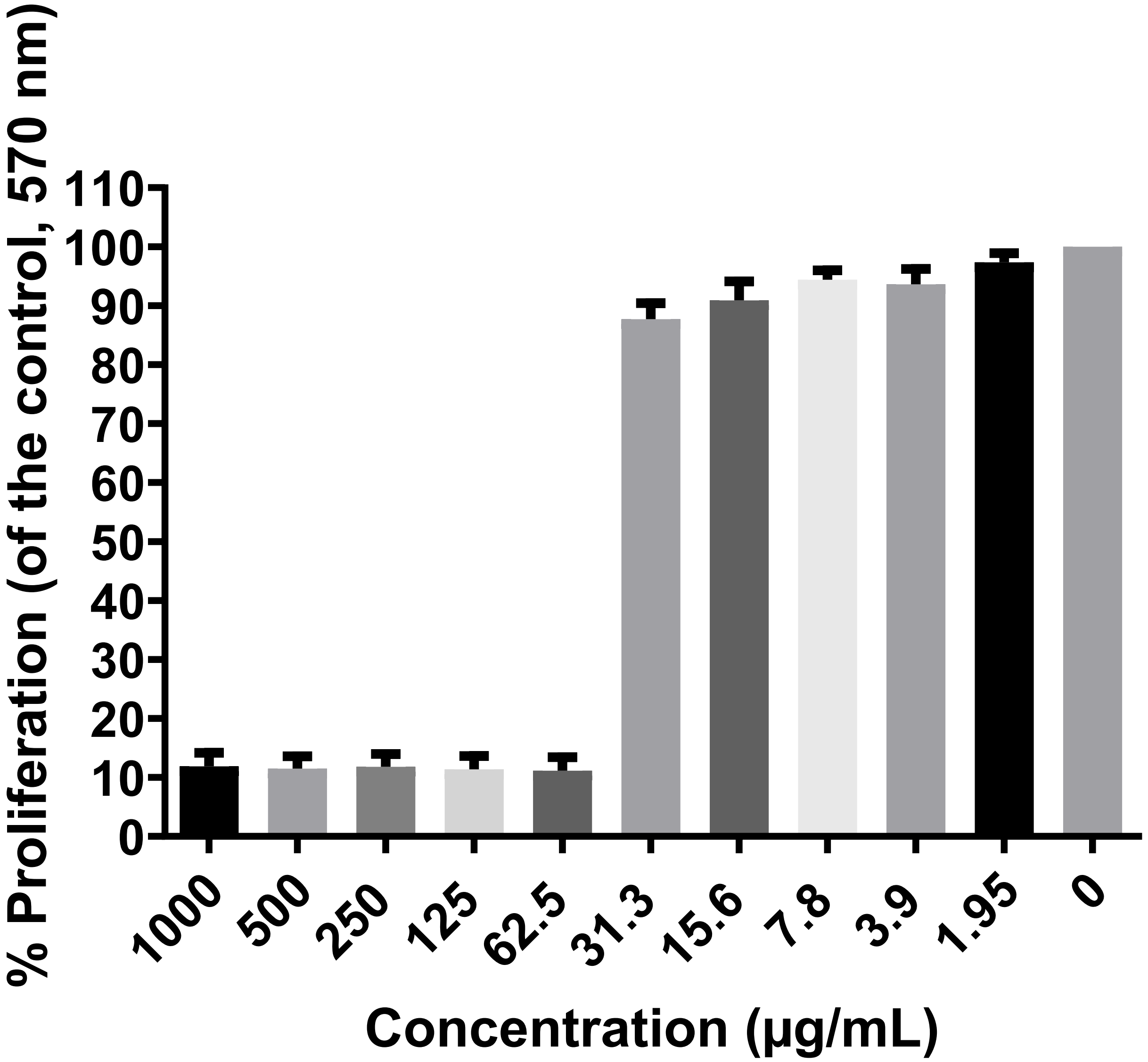

Figure S4. *Dose-Dependent Inhibition of MRSA XIII Proliferation by Palmitoleic Acid*. MRSA XIII cultures were treated with increasing palmitoleic acid concentrations for 18-20 hours at 37 °C. Bacterial proliferation was quantified via the MTT assay and expressed as a percentage of the untreated control (0 µg/mL) at 570 nm. Bars represent the mean ± SEM from six independent biological replicates. A marked dose-dependent reduction in viability was observed, with the minimum inhibitory concentration (MIC) determined to be 62.5 µg/mL under these conditions. This MIC value was subsequently used for the experimental design of biochemical assays.

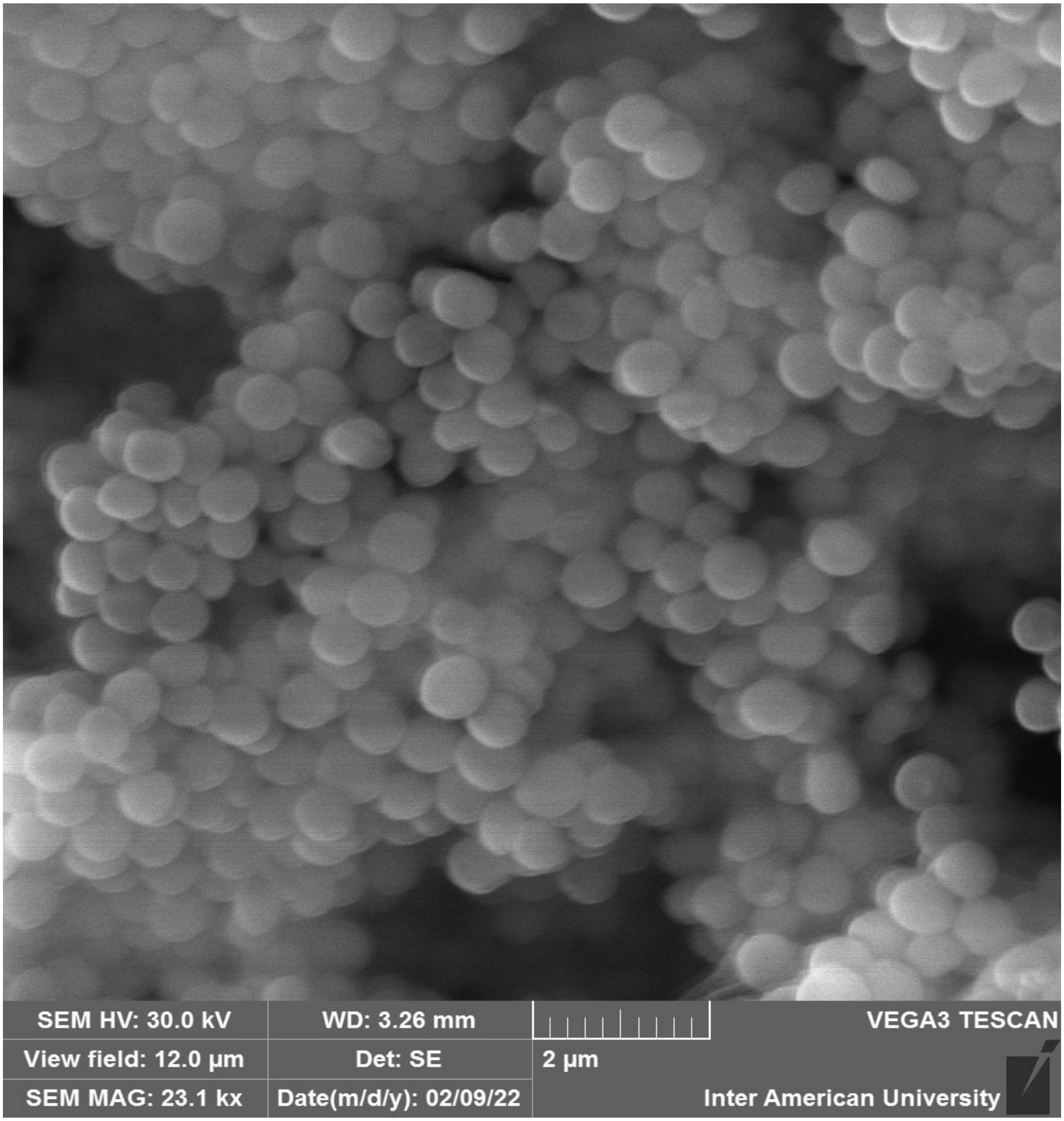

Figure S5. *Scanning Electron Micrograph of MRSA XIII Treated with 1% DMSO (Vehicle Control)*. Representative SEM image of MRSA XIII following treatment with 1% DMSO for 18-20 hours at 37 °C. Image captured using a VEGA3 TESCAN SEM at 30.0 kV with a secondary electron (SE) detector; scale bar = 2 µm.

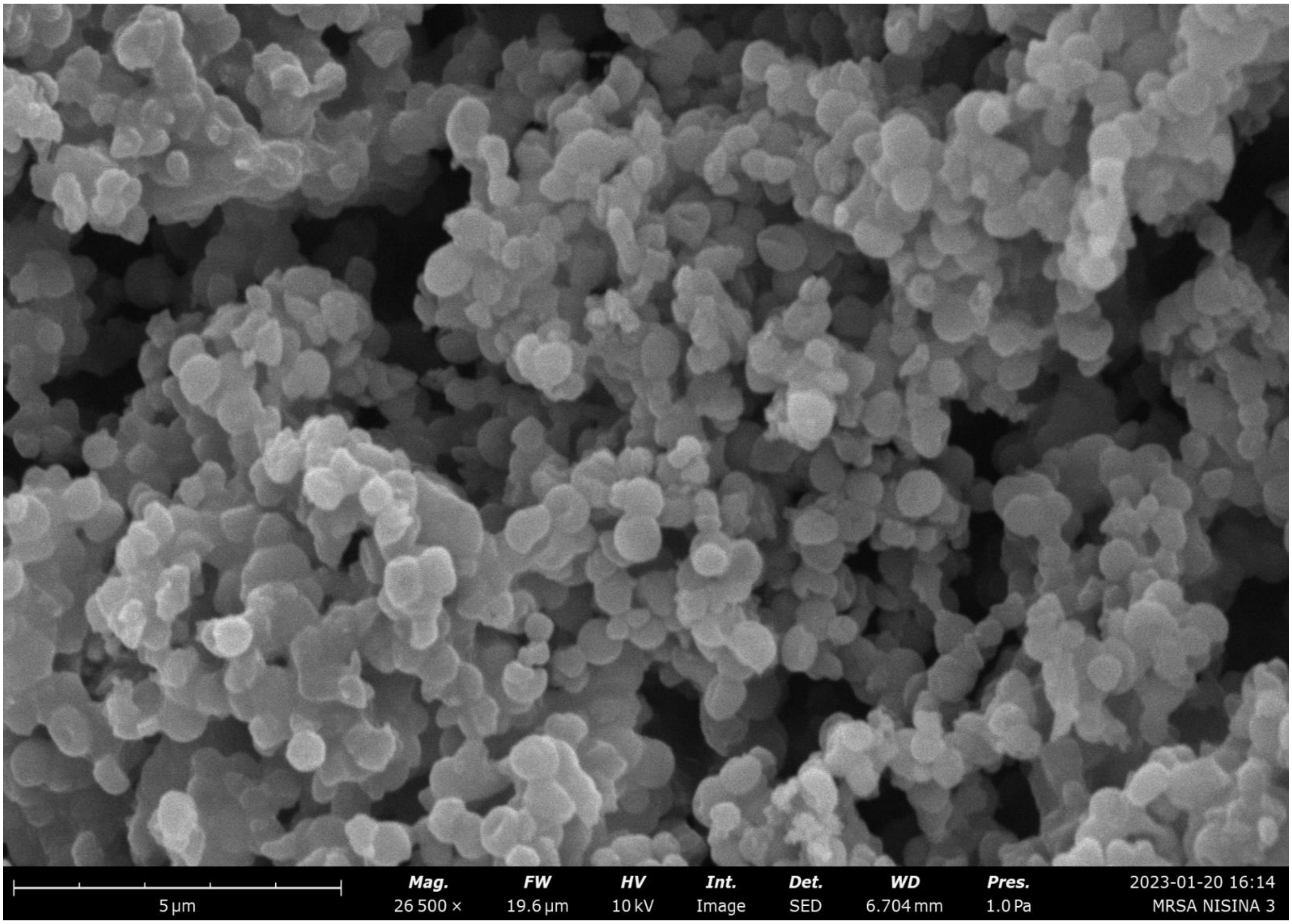

Figure S6. *Scanning Electron Micrograph of MRSA XIII Treated with Nisin*. Representative SEM image of MRSA XIII following treatment with Nisin for 18-20 h at 37 °C. Image captured using a Phemon XL SEM at 10.0 kV with a secondary electron (SE) detector; scale bar = 5 µm.

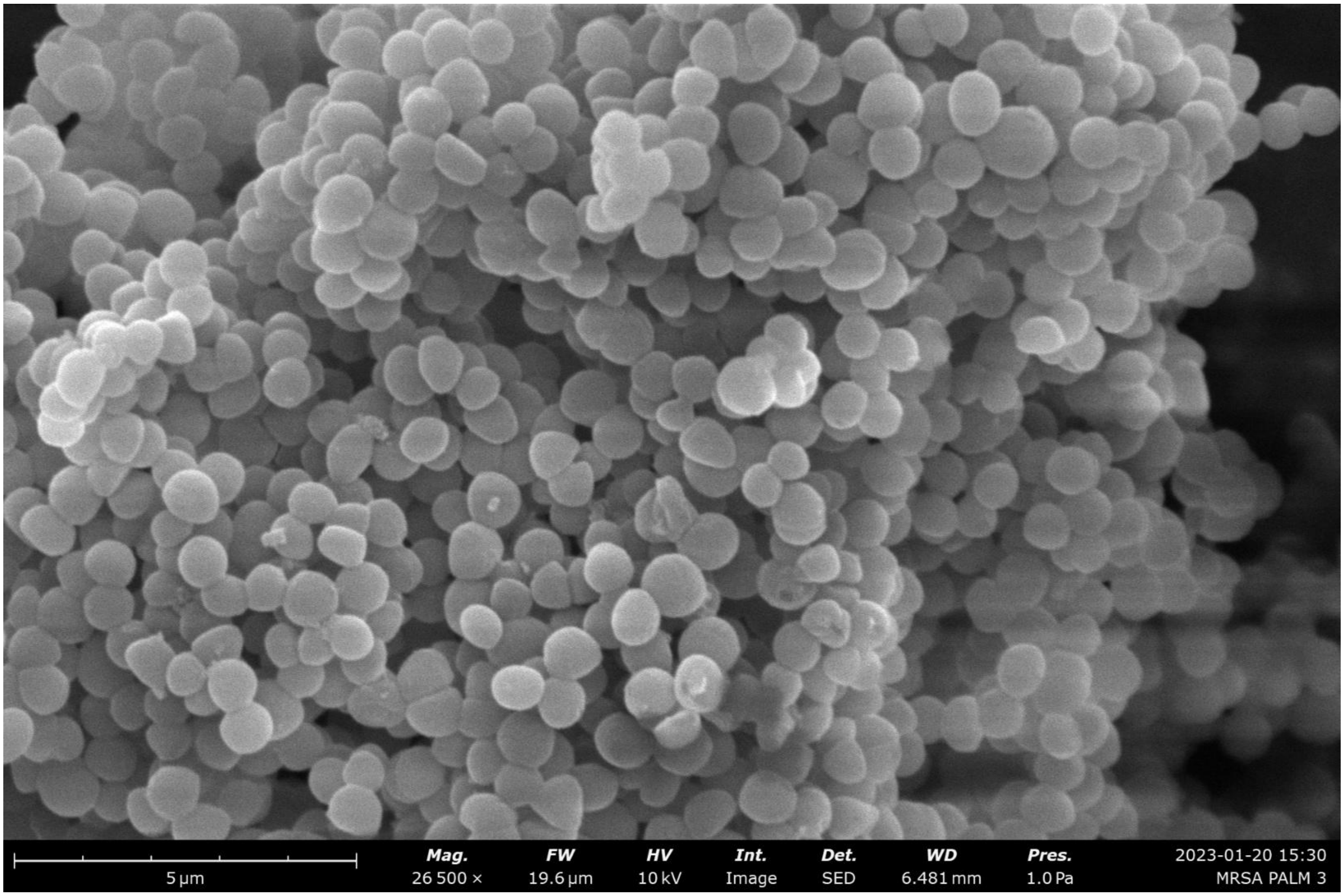

Figure S7. *Scanning Electron Micrograph of MRSA XIII Treated with Palmitoleic Acid*. SEM image of MRSA XIII following exposure to palmitoleic acid. Image captured using a Phemon XL SEM at 10.0 kV with a secondary electron (SE) detector; scale bar = 5 µm.

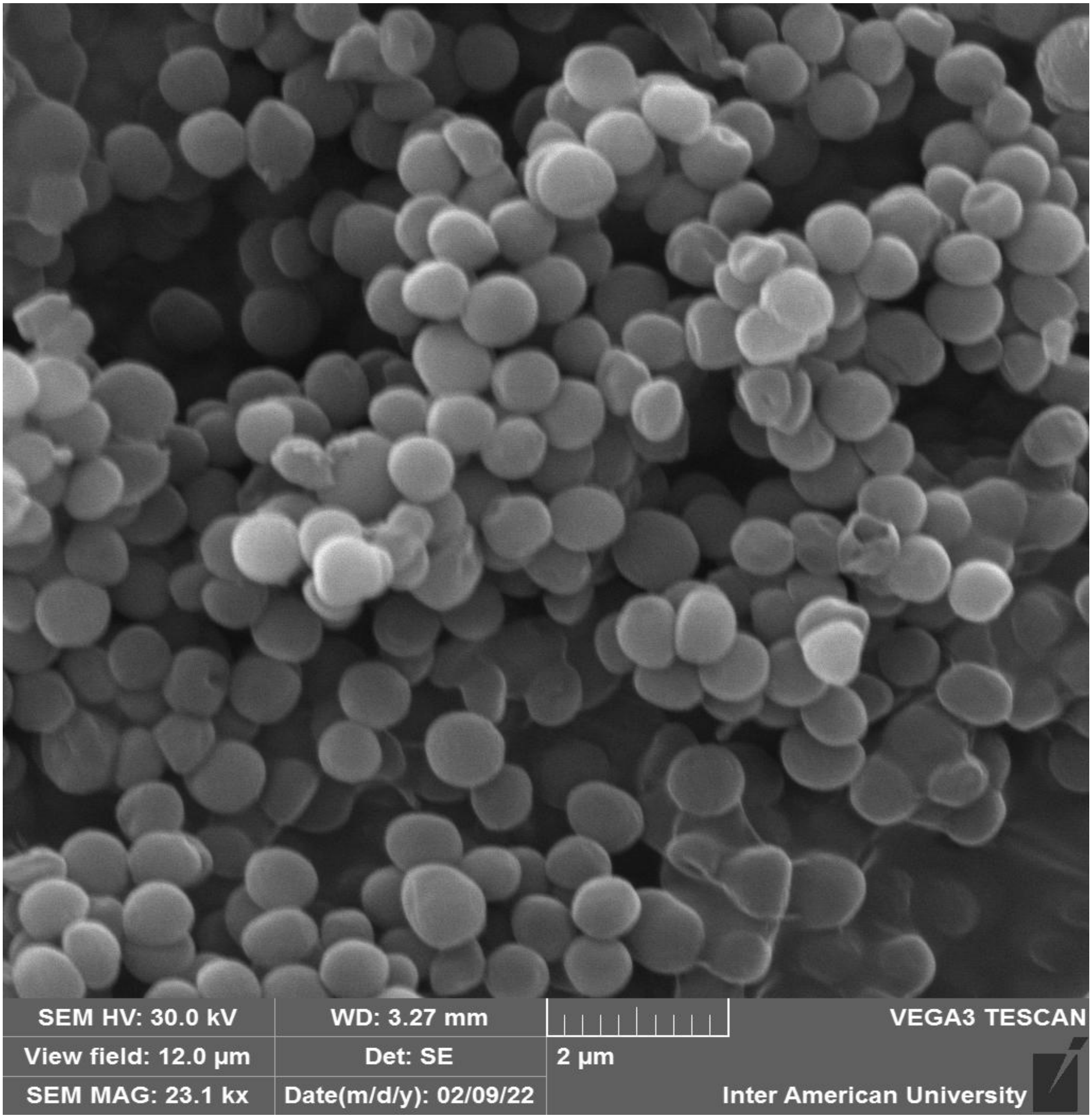

Figure S8. *Scanning Electron Microscopy image of MRSA XIII following treatment with 2-HDA*.Image captured using a VEGA 3 TESCAN SEM at 30.0 kV with a secondary electron (SE) detector; scale bar = 2 μm.

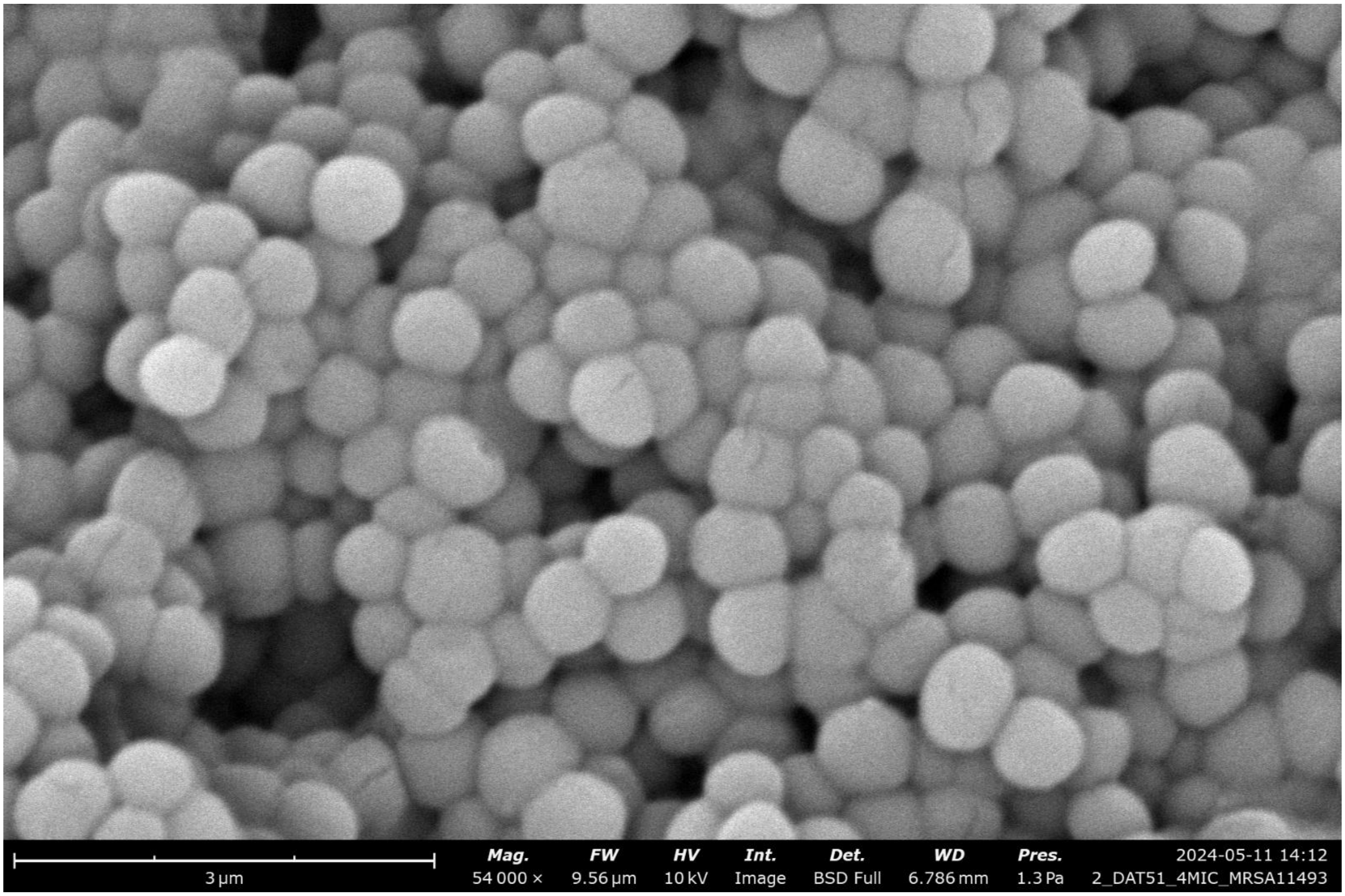

Figure S9. *Scanning Electron Micrograph of MRSA XIII Treated with DAT-51*. SEM image of MRSA XIII following exposure to DAT-51 for 18-20 h at 37 °C. Image captured using a Phenom XL SEM at 10.0 kV with a backscatter electron detector (BSD); scale bar = 3 µm.

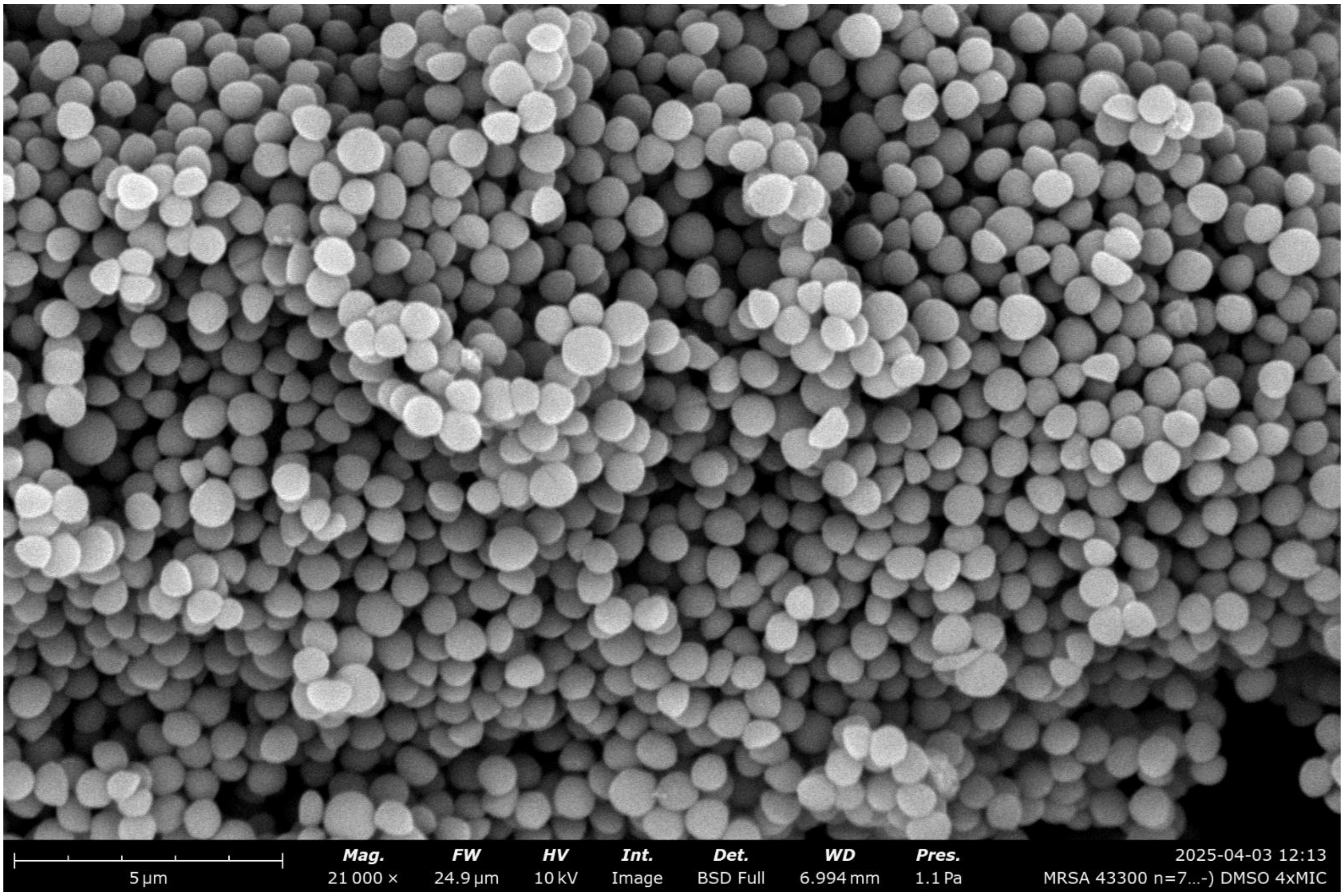

Figure S10. *Scanning Electron Micrograph of MRSA ATCC 43300 Treated with 1% DMSO (Vehicle Control)*. SEM image of MRSA ATCC 43300 following exposure to 1% DMSO for 18-20 h at 37 °C. Image captured using a Phenom XL SEM at 10.0 kV with a backscatter electron detector (BSD); scale bar = 5 µm.

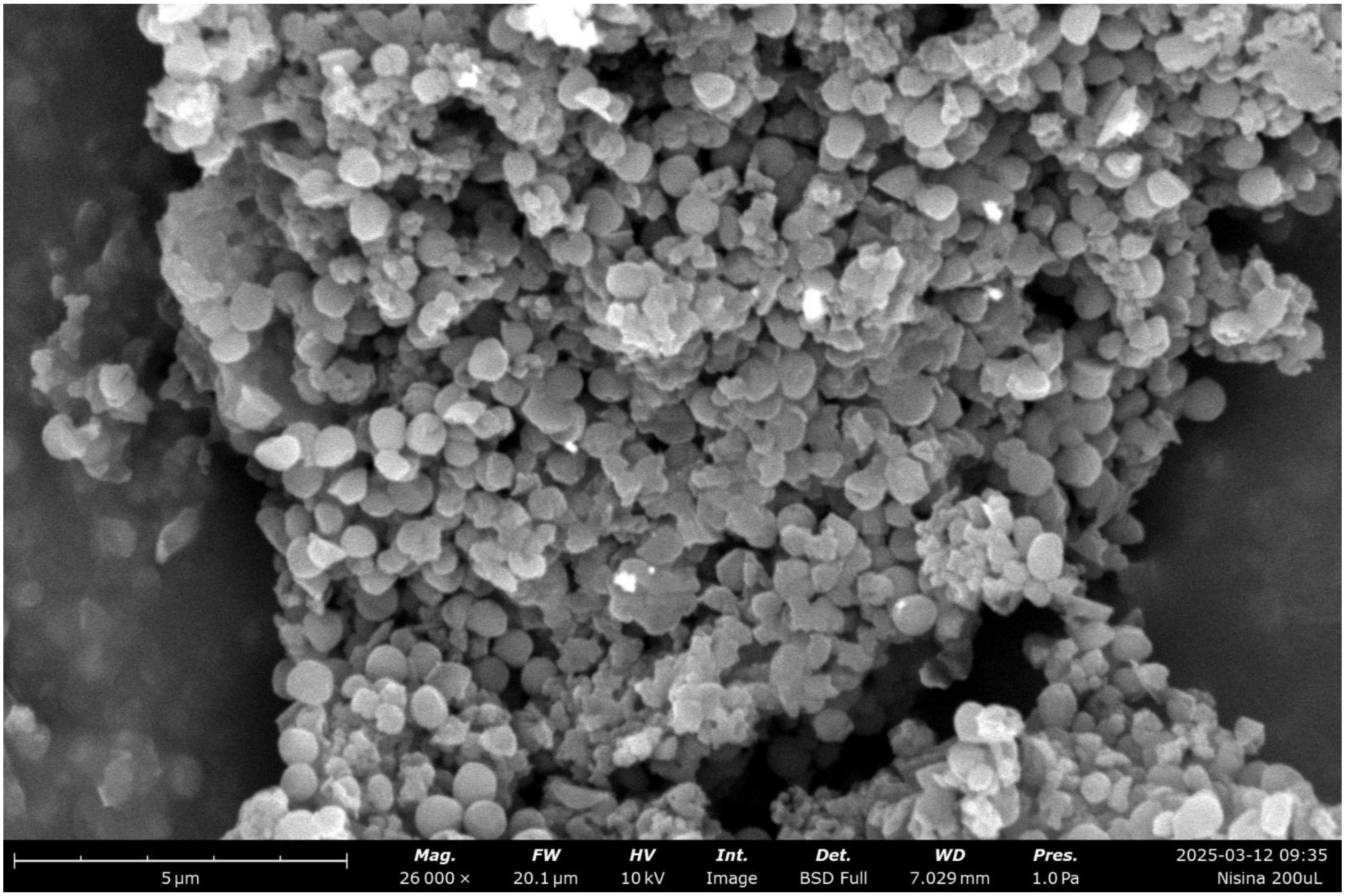

Figure S11. *Scanning Electron Micrograph of MRSA ATCC 43300 Treated with Nisin.* SEM image of MRSA ATCC 43300 following exposure to Nisin for 18-20 h at 37 °C. Image captured using a Phenom XL SEM at 10.0 kV with a backscatter electron detector (BSD); scale bar = 5 µm.

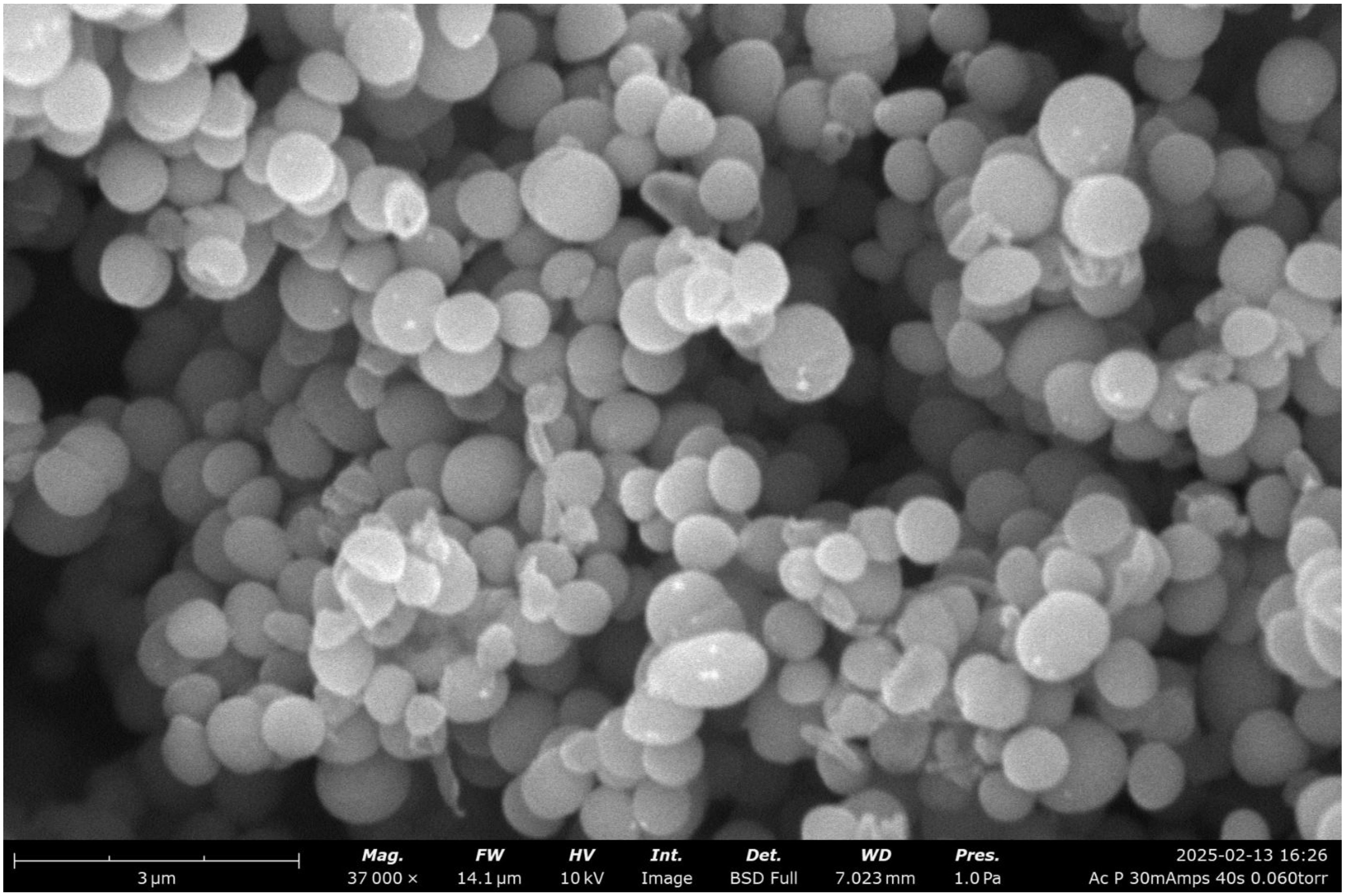

Figure S12. *Scanning Electron Micrograph of MRSA ATCC 43300 Treated with Palmitoleic Acid.* SEM image of MRSA ATCC 43300 following exposure to Palmitoleic Acid for 18-20 h at 37 °C. Image captured using a Phenom XL SEM at 10.0 kV with a backscatter electron detector (BSD); scale bar = 3 µm..

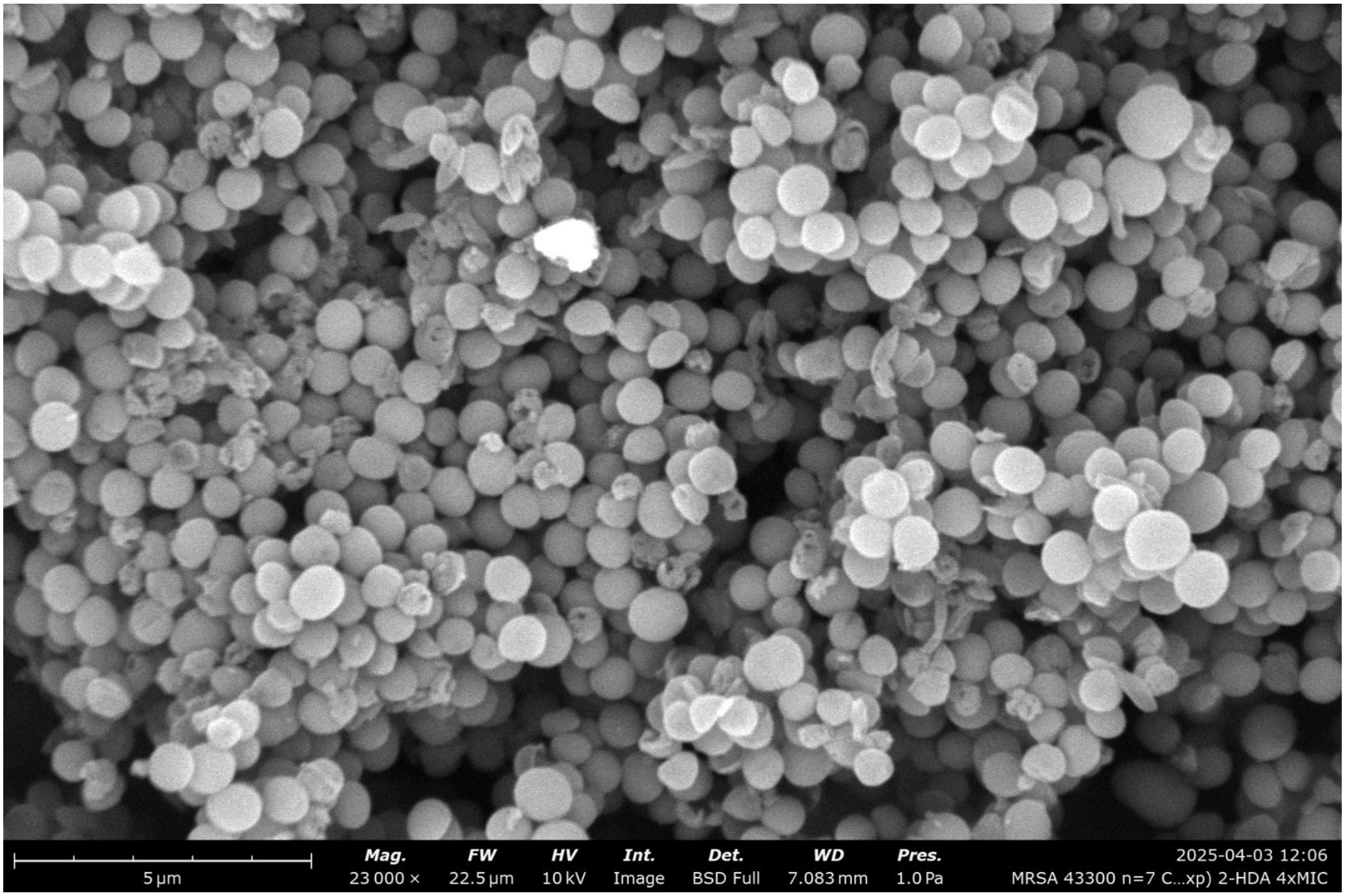

Figure S13. *Scanning Electron Micrograph of MRSA ATCC 43300 Treated with 2-HDA.* SEM image of MRSA ATCC 43300 following exposure to 2-HDA for 18-20 h at 37 °C. Image captured using a Phenom XL SEM at 10.0 kV with a backscatter electron detector (BSD); scale bar = 5 µm.

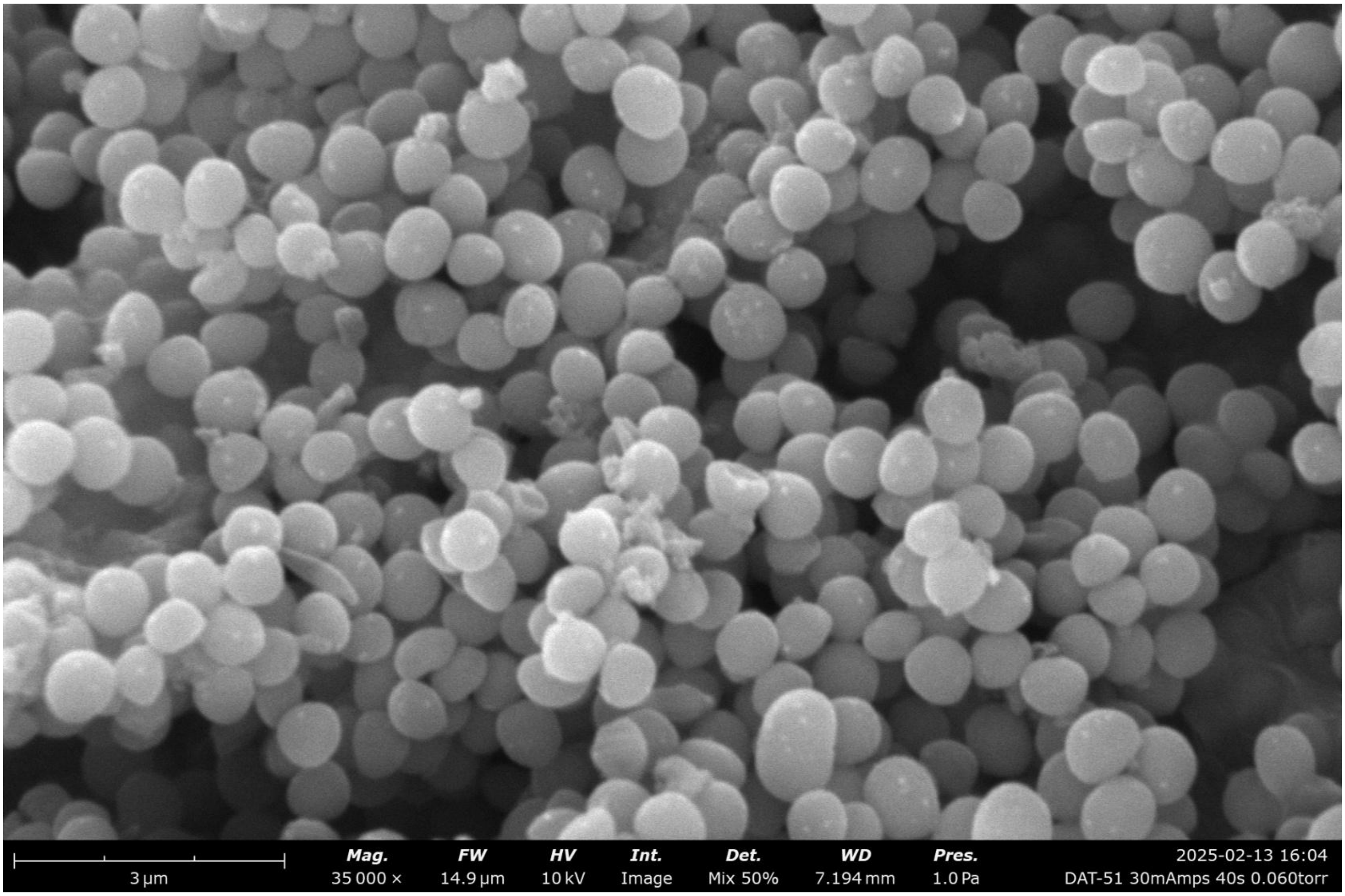

Figure S14. *Scanning Electron Micrograph of MRSA ATCC 43300 Treated with DAT-51.* SEM image of MRSA ATCC 43300 following exposure to DAT-51 for 18-20 h at 37 °C. Image captured using a Phenom XL SEM at 10.0 kV with a mix of backscatter electron detector (BSD) and secondary electron (SE) detector; scale bar = 3 µm.

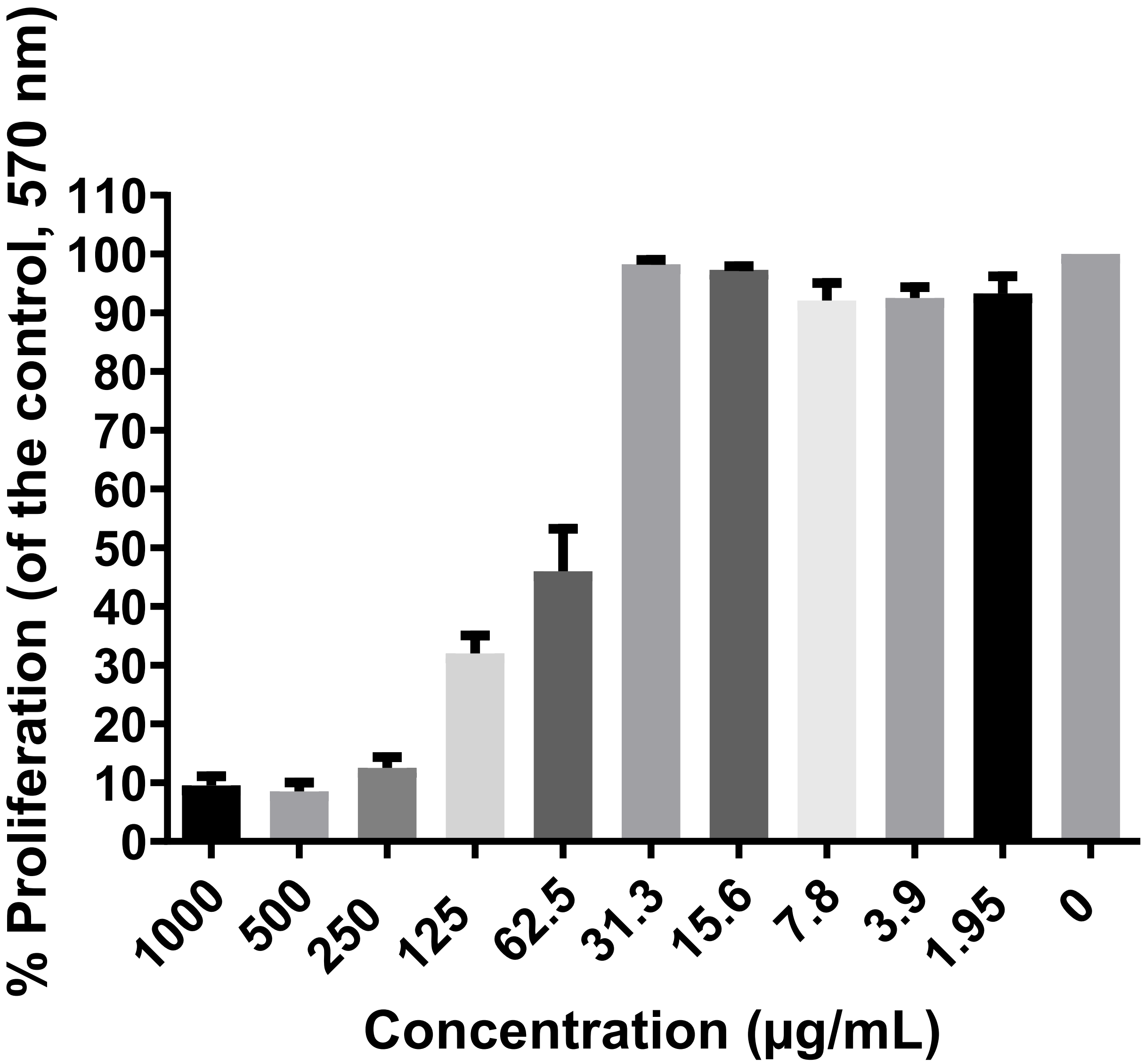

Figure S15. *Dose-Dependent Inhibition of MRSA XIII Proliferation by Linoleic Acid*. MRSA XIII cultures were treated with increasing linoleic acid concentrations for 18-20 hours at 37 °C. Bacterial proliferation was quantified via the MTT assay and expressed as a percentage of the untreated control (0 µg/mL) at 570 nm. Bars represent the mean ± SEM from seven independent biological replicates. A marked dose-dependent reduction in viability was observed, with the minimum inhibitory concentration (MIC) determined to be 62.5 µg/mL under these conditions. This MIC value was subsequently used for the experimental design of biochemical assays.

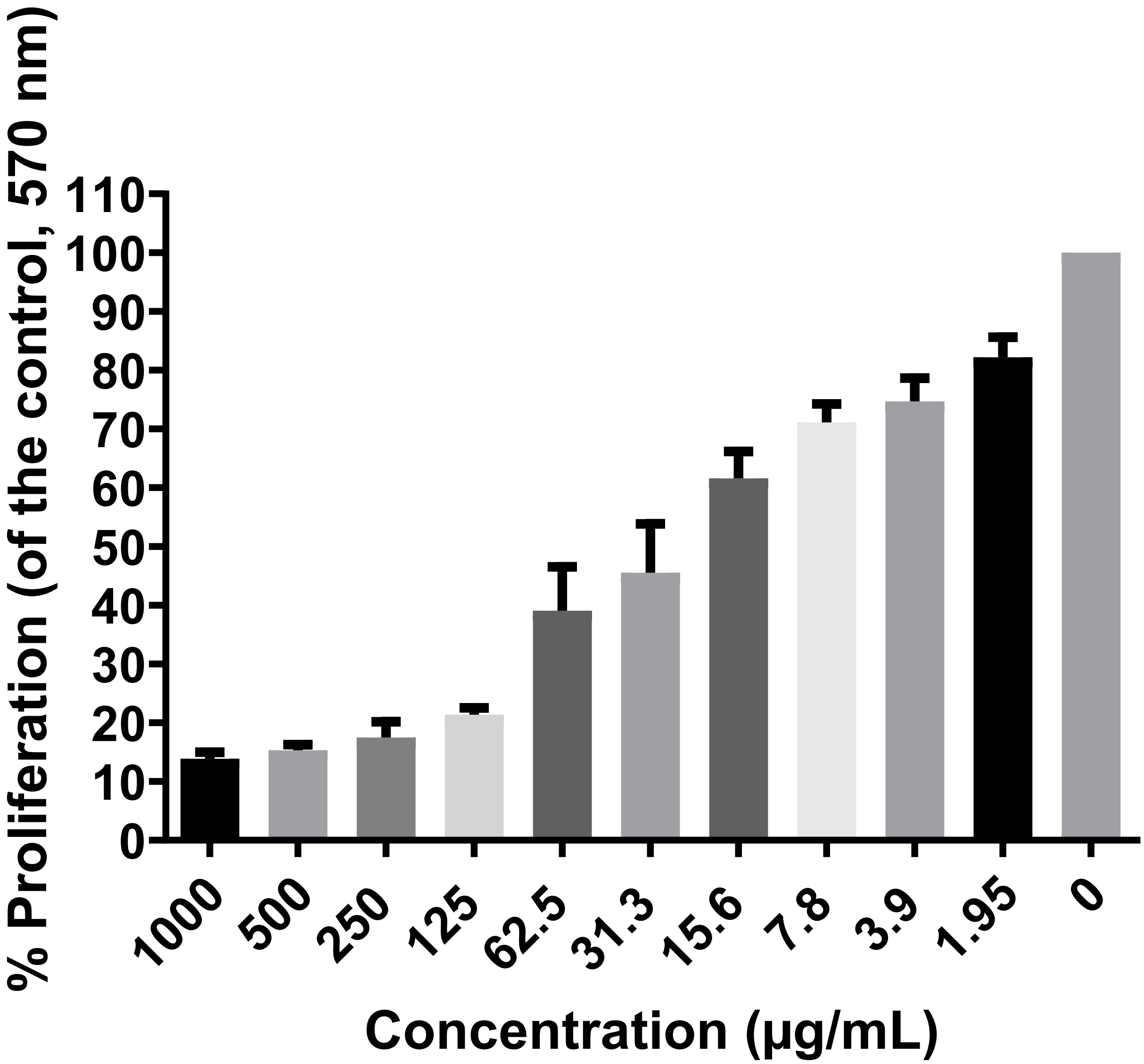

Figure S16. *Dose-Dependent Inhibition of MRSA XIII Proliferation by Phosphomycin*. MRSA XIII cultures were treated with increasing concentrations of phosphomycin for 18-20 hours at 37 °C. Bacterial proliferation was quantified via the MTT assay and expressed as a percentage of the untreated control (0 µg/mL) at 570 nm. Bars represent the mean ± SEM from six independent biological replicates. A marked dose-dependent reduction in viability was observed, with the minimum inhibitory concentration (MIC) determined to be 125 µg/mL under these conditions. This MIC value was subsequently used for the experimental design of MurA inhibition assays.

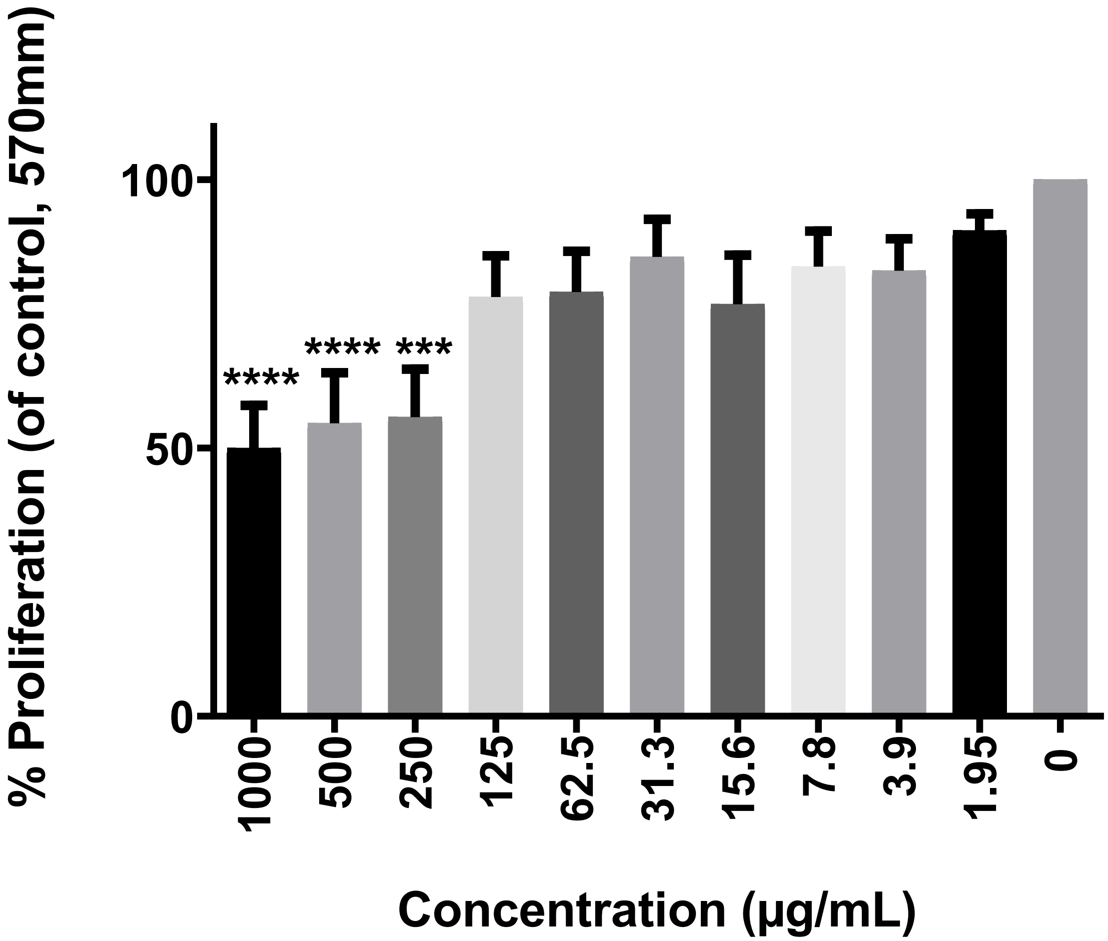

Figure S17. *Evaluation of 2-HDA Cytotoxicity in Vero Cells Using MTT Assay*. Bars represent normalized mean absorbance values at 570 nm ± SEM from six independent biological replicates. One-way ANOVA assessed statistical significance with Dunnett's post hoc test. ****$p < 0.0001$.
